# Supplementary material for: Market, power, gift, and concession economies: Comparison using four-mode primitive network models
Source: PLoS One. 2025 Aug 12;20(8):e0330174. doi: 10.1371/journal.pone.0330174 (PMC12342310; doi:10.1371/journal.pone.0330174)
Supplement: S1 Code — (PDF) [file pone.0330174.s001.pdf]

# Code

Example codes in Mathematica® to calculate the network models corresponding to the four economic modes, Watts–Strogatz, Watts–Strogatz derivative, and Barabási–Albert models.

**(a) Market ecomomy**

```
(*initial setting----------------------------------------------------------------------------------------------------*)
n = 100(*number of nodes*); tmax = 600(*maximum steps*); kmean = 4(*mean degree*);
m0 = Table[0,{i, 1, n}, {j, 1, n}](*adjacency matrix of network at t=0*);
m = Table[m0, {i, 1, tmax}](*adjacency matrix from t=1 to tmax*);
g0 = AdjacencyGraph[m0, DirectedEdges -> True](*network graph at t=0*);
g = Table[AdjacencyGraph[m0, DirectedEdges -> True], {i, 1, tmax}];(*network graph from t=1 to tmax*)
nodelist = Table[i, {i, 1, n}](*node list*);

(*calculation of network from t=2 to tmax-------------------------------------------------------------------*)
Do[
  m[[t]] = m[[t - 1]]; g[[t]] = g[[t - 1]];
  node = RandomChoice[nodelist, 2](*random selection of two nodes*);
  nodelist = Join[nodelist, node](*add two selected nodes*);
  nodelist = Take[nodelist, -n](*delete two unselected nodes*);
  If[node[[1]] == node[[2]],
    m[[t]] = m[[t]](*remove self-loop*),
    m[[t]] = ReplacePart[m[[t]],
      {{node[[1]], node[[2]]} -> 1, {node[[2]], node[[1]]} -> 1}]](*generation of bidirectional edges*);
  g[[t]] = AdjacencyGraph[m[[t]], DirectedEdges -> True];
  If[EdgeCount[g[[t]]]/n <= kmean,
    g[[t]] = g[[t]](*mean degree is kmean or less*),
    po = Position[m[[t]], 1]; co = Count[po, {u_, _} /; u > 0];
    ra = RandomInteger[{1, co}]; ta = Take[po, {ra}];
    m[[t]] = ReplacePart[m[[t]],
      {{ta[[1]][[1]], ta[[1]][[2]]} -> 0, {ta[[1]][[2]], ta[[1]][[1]]} -> 0}](*delete edges*);
    g[[t]] = AdjacencyGraph[m[[t]], DirectedEdges -> True](*mean degree exceeds kmean*),
{t, 2, tmax}]

(*drawing network graph-------------------------------------------------------------------------------------*)
Graph[g[[t]], DirectedEdges -> True, GraphLayout -> "SpringElectricalEmbedding"]

(*calculation and drawing of network features------------------------------------------------------------*)
ListLinePlot[{
  Table[MeanClusteringCoefficient[g[[i]]], {i, 1, tmax}],
```

```
  Table[GraphDensity[g[[i]]], {i, 1, tmax}]}]
ListLinePlot[{
  Table[GraphReciprocity[g[[i]]], {i, 1, tmax}],
  Table[GraphAssortativity[g[[i]]], {i, 1, tmax}]}]
ListLinePlot[{
  Table[Mean[ClosenessCentrality[g[[i]]]], {i, 1, tmax}],
  Table[StandardDeviation[ClosenessCentrality[g[[i]]]], {i, 1, tmax}]}]

(*calculation of Gini coefficient-----------------------------------------------------------------------------*)
mtmax= m[[tmax]]; mprop = m[[tmax]](*initial setting*);
scol = Table[0, {j, 1, n}](*initial setting*);
Do[scol[[j]] = Total[mtmax[[All, j]]], {j, 1, n}](*element sum in column of matrix*);
(*calculation of propagation matrix*)
Do[
  Do[
    If[scol[[j]] == 0,
      x = mtmax[[i, j]];
      mprop = ReplacePart[mprop, {i, j} -> x],
      x = mtmax[[i, j]]/scol[[j]](*propagation ratio*);
      mprop = ReplacePart[mprop, {i, j} -> x]],
  {j, 1, n}],
{i, 1, n}]
(*setting column vector*)
v0 = Table[0, {i, n}, {1}]; vn = RandomChoice[nodelist];
v0 = ReplacePart[v0, {vn, 1} -> 1];
(*calculation of Gini coefficient*)
tprop = 100(*propagation time*);
vt = Table[v0, {t, 1, tprop}]; gini = Table[0, tprop](*initial setting*);
gsort = Sort[vt[[1]]];
gini[[1]] = (2*Sum[i*Total[gsort[[i]]], {i, 1, n}])/(n*Sum[Total[gsort[[i]]], {i, 1, n}]) - (n + 1)/n;
Do[
  vt[[t]] = mprop.vt[[t - 1]];
  gsort = Sort[vt[[t]]];
  gini[[t]] = (2*Sum[i*Total[gsort[[i]]], {i, 1, n}])/(n*Sum[Total[gsort[[i]]], {i, 1, n}]) - (n + 1)/n,
{t, 2, tprop}]
(*drawing of Gini coefficient*)
ListLogLinearPlot[Table[gini[[t]], {t, 1, tprop}]]
```

**(b) Power economy**

Codes different from (a) market economy.

```
(*calculation of network from t=2 to tmax----------------------------------------------------------------*)
Do[
    m[[t]] = m[[t - 1]]; g[[t]] = g[[t - 1]];
    node = RandomChoice[nodelist, 2](*random selection of two nodes*);
    nodelist = Join[nodelist, node](*add two selected nodes*);
    If[node[[1]] == node[[2]],
        m[[t]] = m[[t]](*remove self-loop*),
        m[[t]] = ReplacePart[m[[t]],
          {{node[[1]], node[[2]]} -> 1, {node[[2]], node[[1]]} -> 1}]](*generation of bidirectional edges*);
    g[[t]] = AdjacencyGraph[m[[t]], DirectedEdges -> True];
    If[EdgeCount[g[[t]]]/n <= kmean,
        g[[t]] = g[[t]](*mean degree is kmean or less*),
        po = Position[m[[t]], 1]; co = Count[po, {u_, _} /; u > 0];
        ra = RandomInteger[{1, co}]; ta = Take[po, {ra}];
        m[[t]] = ReplacePart[m[[t]],
           {{ta[[1]][[1]], ta[[1]][[2]]} -> 0, {ta[[1]][[2]], ta[[1]][[1]]} -> 0}](*delete edges*);
        g[[t]] = AdjacencyGraph[m[[t]], DirectedEdges -> True](*mean degree exceeds kmean*),
{t, 2, tmax}]
```

**(c) Gift economy**

Codes different from (a) market economy.

```
(*calculation of network from t=2 to tmax----------------------------------------------------------------*)
Do[
    m[[t]] = m[[t - 1]]; g[[t]] = g[[t - 1]];
    node = RandomChoice[nodelist, 2](*random selection of two nodes*);
    If[node[[1]] == node[[2]],
        m[[t]] = m[[t]](*remove self-loop*),
        m[[t]] = ReplacePart[m[[t]],
          {{node[[1]], node[[2]]} -> 1, {node[[2]], node[[1]]} -> 1}]](*generation of bidirectional edges*);
    g[[t]] = AdjacencyGraph[m[[t]], DirectedEdges -> True];
    If[EdgeCount[g[[t]]]/n <= kmean,
        g[[t]] = g[[t]](*mean degree is kmean or less*),
        po = Position[m[[t]], 1]; co = Count[po, {u_, _} /; u > 0];
        ra = RandomInteger[{1, co}]; ta = Take[po, {ra}];
        m[[t]] = ReplacePart[m[[t]],
           {{ta[[1]][[1]], ta[[1]][[2]]} -> 0, {ta[[1]][[2]], ta[[1]][[1]]} -> 0}](*delete edges*);
        g[[t]] = AdjacencyGraph[m[[t]], DirectedEdges -> True](*mean degree exceeds kmean*),
{t, 2, tmax}]
```

**(d) Concession economy**

Codes different from (a) market economy.

```
(*initial setting------match the edge generation speed and disappearance speed to others------------*)
n = 100(*number of nodes*); tmax =600*2(*2 counts per step*); tconst = 400(*time constant*);
nodehistory = {}(*node list*);

(*calculation of network from t=2 to tmax--------------------------------------------------------------*)
Do[
  m[[t]] = m[[t - 1]]; g[[t]] = g[[t - 1]];
  node = RandomChoice[nodelist, 2](*random selection of two nodes*);
  nodehistory = Append[nodehistory, node](*add two selected nodes*);
  If[node[[1]] == node[[2]],
    m[[t]] = m[[t]](*remove self-loop*),
    m[[t]] = ReplacePart[m[[t]],
      {{node[[1]], node[[2]]} -> 1]](*generation of unidirectional edge*);
  g[[t]] = AdjacencyGraph[m[[t]], DirectedEdges -> True];
  If[t <= tconst,
    g[[t]] = g[[t]](*time is tconst or less*),
    ta = Take[nodehistory, {t - tconst}](*take oldest nodes*);
    m[[t]] = ReplacePart[m[[t]],
      {ta[[1]][[1]], ta[[1]][[2]]} -> 0](*delete oldest edge*);
    g[[t]] = AdjacencyGraph[m[[t]], DirectedEdges -> True](*time exceeds tconst*),
{t, 2, tmax}]
```

**(e) Watts–Strogatz model (bidireiction)**

Codes different from (a) market economy.

```
(*initial setting-------------------------------------------------------------------------------------*)
n = 100(*number of nodes*);
kws = 2(*degree per node*);  ρ  = 0.7(*rewiring probability*);

(*calculation of network------------------------------------------------------------------------------*)
g = RandomGraph[WattsStrogatzGraphDistribution[n,  ρ  kws]](*undirected graph*);
m = AdjacencyMatrix[g] (*adjacency matrix*);
g = AdjacencyGraph[m, DirectedEdges -> True](*bidirectional graph*);
```

**(f) Watts–Strogatz derivative model (unidirection)**

Codes different from (a) market economy.

```
(*initial setting-------------------------------------------------------------------------------------*)
n = 100(*number of nodes*);
kws = 2(*degree per node*);  ρ  = 0.7(*rewiring probability*); rewire = 0;
```

```
(*calculation of network------------------------------------------------------------------------------------------*)
g0 = RandomGraph[WattsStrogatzGraphDistribution[n, 0, kws]](*undirected graph*);
m0 = AdjacencyMatrix[g0](*adjacency matrix*); m = m0;
Do[
  Do[
    If[m0[[i]][[j]] == 0,
      m = m,
      node = RandomSample[nodelist, 2];
      m = ReplacePart[m, {i, j} -> 0];
      m = ReplacePart[m, {node[[1]], node[[2]]} -> 1];
      rewire = rewire + 1];
      If[rewire > n*kws*ρ*2, Break[]],
  {j, 1, n}],
{i, 1, n}];
g = AdjacencyGraph[m, DirectedEdges -> True](*unidirectional graph*)
```

**(g) Barabási–Albert model**

Codes different from (a) market economy.

```
(*calculation of network from t=2 to tmax------------------------------------------------------------------*)
Do[
  m[[t]] = m[[t - 1]]; g[[t]] = g[[t - 1]];
  node = RandomSample[Table[VertexDegree[g[[t]], nodelist[[i]]], {i, 1, n}] + 2 -> nodelist, 2](*random selection of
  two nodes proportional to degree*);
  If[node[[1]] == node[[2]],
    m[[t]] = m[[t]](*remove self-loop*),
    m[[t]] = ReplacePart[m[[t]],
      {{node[[1]], node[[2]]} -> 1, {node[[2]], node[[1]]} -> 1}]](*generation of bidirectional edges*);
  g[[t]] = AdjacencyGraph[m[[t]], DirectedEdges -> True];
  If[EdgeCount[g[[t]]]/n <= kmean,
    g[[t]] = g[[t]](*mean degree is kmean or less*),
    po = Position[m[[t]], 1]; co = Count[po, {u_, _} /; u > 0];
    ra = RandomInteger[{1, co}]; ta = Take[po, {ra}];
    m[[t]] = ReplacePart[m[[t]],
      {{ta[[1]][[1]], ta[[1]][[2]]} -> 0, {ta[[1]][[2]], ta[[1]][[1]]} -> 0}](*delete edges*);
    g[[t]] = AdjacencyGraph[m[[t]], DirectedEdges -> True](*mean degree exceeds kmean*),
{t, 2, tmax}]
```
