# Supplementary material for: Market, power, gift, and concession economies: Comparison using four-mode primitive network models
Source: PLoS One. 2025 Aug 12;20(8):e0330174. doi: 10.1371/journal.pone.0330174 (PMC12342310; doi:10.1371/journal.pone.0330174)
Supplement: S1 Data — (PDF) [file pone.0330174.s002.pdf]

Data

(a) Market ecomomy

	C	D	R	S	c_μ	c_σ	G
1	0.110147	0.020606	1	0.076367	0.152239	0.198965	0.779706
2	0.130278	0.022828	1	-0.031997	0.161666	0.238151	0.811150
3	0.116232	0.022424	1	0.099734	0.181931	0.244128	0.791091
4	0.155560	0.026061	1	-0.000298	0.173331	0.238563	0.788527
5	0.106088	0.015960	1	-0.209436	0.137525	0.218134	0.840759
6	0.126287	0.025455	1	0.007126	0.188789	0.255407	0.793760
7	0.112444	0.020404	1	0.113902	0.150033	0.221699	0.807921
8	0.149352	0.020202	1	-0.234727	0.159072	0.235119	0.828300
9	0.120451	0.022222	1	-0.042858	0.159544	0.230205	0.809091
10	0.132991	0.026263	1	0.032861	0.163625	0.231689	0.797846
11	0.150478	0.024647	1	-0.101577	0.166363	0.250661	0.813115
12	0.135161	0.023232	1	-0.000069	0.172141	0.257959	0.814649
13	0.128145	0.018586	1	-0.171092	0.149171	0.221478	0.831196
14	0.123673	0.022222	1	0.200791	0.172451	0.242981	0.809174
15	0.128628	0.023838	1	0.096174	0.162809	0.212597	0.781695
16	0.124988	0.019596	1	0.035973	0.176778	0.238744	0.801458
17	0.110129	0.018990	1	0.133809	0.138003	0.205334	0.815319
18	0.127817	0.022424	1	0.189846	0.190668	0.269143	0.813333
19	0.122427	0.020808	1	0.041102	0.144024	0.209786	0.812524
20	0.134213	0.021212	1	-0.220611	0.178604	0.218389	0.793714
21	0.124390	0.023232	1	-0.036909	0.190121	0.249428	0.801930
22	0.146305	0.020606	1	-0.070076	0.173143	0.263727	0.829010
23	0.132057	0.024444	1	0.077264	0.179828	0.254097	0.798583
24	0.148138	0.025455	1	0.162446	0.163559	0.219134	0.800397
25	0.123443	0.018788	1	-0.138368	0.155068	0.218842	0.798925
26	0.119182	0.024040	1	0.050711	0.209067	0.274399	0.792222
27	0.121468	0.022222	1	-0.156130	0.163367	0.230355	0.801818
28	0.159833	0.022828	1	0.053114	0.184803	0.246865	0.794375
29	0.092069	0.020808	1	0.119454	0.154674	0.206678	0.802913
30	0.100010	0.018990	1	0.017779	0.148394	0.224123	0.824468
31	0.103441	0.019394	1	-0.080103	0.170233	0.242589	0.808526
32	0.120638	0.020202	1	-0.081191	0.152408	0.230262	0.825200
33	0.122121	0.027071	1	0.150353	0.181334	0.218264	0.771716
34	0.105694	0.020808	1	0.109902	0.149476	0.220765	0.804757
35	0.145893	0.023030	1	-0.067506	0.190814	0.261234	0.792212
36	0.117707	0.023636	1	0.109657	0.177809	0.256356	0.809655
37	0.108256	0.023434	1	0.242981	0.172706	0.249834	0.792348
38	0.130035	0.021818	1	0.139022	0.173679	0.236417	0.797477
39	0.121036	0.020000	1	-0.088947	0.163889	0.209587	0.800606
40	0.108408	0.024444	1	0.085737	0.204688	0.274473	0.794622
41	0.136836	0.026263	1	-0.014924	0.171096	0.232066	0.791615
42	0.108631	0.017172	1	0.006551	0.139387	0.219145	0.818118
43	0.126077	0.021414	1	-0.012713	0.194049	0.287149	0.816538
44	0.087860	0.018990	1	-0.004691	0.152545	0.198913	0.788193
45	0.120049	0.024444	1	0.008354	0.192699	0.244474	0.791917
46	0.111260	0.019596	1	-0.095213	0.140717	0.205061	0.820309
47	0.115747	0.020000	1	-0.000587	0.171863	0.237896	0.811224
48	0.112280	0.023232	1	0.141219	0.189510	0.258526	0.793543
49	0.128429	0.017374	1	-0.132648	0.144726	0.219535	0.831395
50	0.130689	0.022626	1	0.019384	0.186118	0.248115	0.782252

(a) Market ecomomy (continued)

	C	D	R	S	c_μ	c_σ	G
51	0.115888	0.023232	1	0.181490	0.176155	0.245316	0.792368
52	0.124025	0.022424	1	0.014596	0.176951	0.250719	0.799818
53	0.107641	0.020404	1	0.085196	0.153713	0.204959	0.797426
54	0.111761	0.026263	1	0.054400	0.201385	0.248968	0.781008
55	0.116469	0.020606	1	-0.082467	0.155004	0.220567	0.819902
56	0.111030	0.025455	1	0.344527	0.181660	0.241481	0.788880
57	0.147129	0.021414	1	-0.008323	0.152490	0.220811	0.806604
58	0.140441	0.024849	1	0.068672	0.213592	0.268294	0.789421
59	0.129988	0.025253	1	0.174348	0.167360	0.217294	0.778960
60	0.116169	0.024849	1	0.155855	0.198628	0.284436	0.804463
61	0.135308	0.021818	1	0.064877	0.189598	0.268877	0.818679
62	0.125197	0.023636	1	0.217775	0.169392	0.242837	0.801898
63	0.110458	0.021616	1	-0.079308	0.163873	0.201491	0.768037
64	0.140117	0.023232	1	-0.061157	0.187966	0.258511	0.799211
65	0.101544	0.020606	1	-0.061515	0.176377	0.257513	0.808713
66	0.114604	0.019394	1	-0.103237	0.181898	0.246915	0.812979
67	0.144490	0.021010	1	-0.013197	0.156660	0.222376	0.814327
68	0.118978	0.019798	1	-0.109351	0.156651	0.221518	0.813163
69	0.125665	0.020808	1	0.150150	0.199274	0.305970	0.827800
70	0.105635	0.021414	1	-0.154757	0.163723	0.230189	0.804906
71	0.137633	0.019798	1	-0.064903	0.148811	0.225558	0.823776
72	0.123413	0.022020	1	0.078199	0.166182	0.216181	0.792752
73	0.119222	0.027071	1	0.076900	0.196601	0.244631	0.780000
74	0.146827	0.023434	1	-0.108064	0.159154	0.235496	0.815776
75	0.097800	0.022424	1	0.042123	0.181882	0.248095	0.797909
76	0.118075	0.016768	1	0.041111	0.151403	0.232886	0.826707
77	0.086943	0.020404	1	0.123938	0.176528	0.242358	0.794000
78	0.126200	0.024242	1	0.231978	0.152209	0.200670	0.790172
79	0.117819	0.023434	1	0.066999	0.178413	0.237474	0.789304
80	0.111636	0.023434	1	0.211155	0.172644	0.253923	0.800783
81	0.110400	0.024040	1	0.253038	0.181293	0.239963	0.774915
82	0.159158	0.027071	1	-0.082814	0.191940	0.271648	0.798195
83	0.112053	0.023636	1	0.350245	0.177892	0.233769	0.785172
84	0.134732	0.026263	1	0.171272	0.184687	0.255518	0.793488
85	0.121575	0.023434	1	0.157017	0.172527	0.247019	0.813304
86	0.129255	0.024647	1	0.022265	0.151070	0.198780	0.801481
87	0.113223	0.019394	1	-0.002401	0.172162	0.250070	0.824842
88	0.126423	0.023434	1	0.099180	0.179859	0.250334	0.806000
89	0.131075	0.024647	1	-0.004771	0.163190	0.225981	0.793934
90	0.107616	0.022626	1	0.175481	0.171564	0.256810	0.811441
91	0.108822	0.020202	1	0.061479	0.170829	0.247144	0.808776
92	0.122591	0.023434	1	0.026643	0.205375	0.271975	0.798509
93	0.113134	0.022020	1	0.202726	0.142456	0.211555	0.813028
94	0.111230	0.018384	1	0.083251	0.202494	0.283469	0.819310
95	0.117445	0.019596	1	-0.254276	0.168296	0.223051	0.812887
96	0.134879	0.025051	1	0.094224	0.191788	0.254777	0.793984
97	0.119635	0.023636	1	-0.009625	0.184604	0.255332	0.805172
98	0.164681	0.024647	1	-0.107390	0.168542	0.238143	0.806066
99	0.138317	0.028283	1	0.249188	0.211328	0.264160	0.783043
100	0.101235	0.022828	1	0.010962	0.183660	0.258352	0.806696

(b) Power economy

	C	D	R	S	c_μ	c_σ	G
1	0.097946	0.040404	1	-0.089146	0.286603	0.152803	0.535100
2	0.084483	0.040404	1	-0.067977	0.286245	0.165157	0.546550
3	0.097701	0.040404	1	-0.191917	0.287207	0.160359	0.528400
4	0.065928	0.040404	1	-0.055988	0.304403	0.164745	0.484975
5	0.117373	0.040404	1	-0.112484	0.290216	0.158284	0.529900
6	0.121275	0.040404	1	-0.118553	0.290627	0.146567	0.512950
7	0.125277	0.040404	1	-0.183690	0.298313	0.148909	0.517450
8	0.080365	0.040404	1	-0.174248	0.325922	0.202523	0.516061
9	0.061695	0.040404	1	-0.051558	0.287952	0.140510	0.511100
10	0.079678	0.040404	1	-0.110487	0.289626	0.127945	0.487550
11	0.116010	0.040404	1	-0.030549	0.286208	0.148906	0.523650
12	0.102197	0.040404	1	-0.024746	0.298500	0.196418	0.549045
13	0.095504	0.040404	1	-0.139123	0.295894	0.139072	0.500300
14	0.095691	0.040404	1	-0.186541	0.292870	0.163970	0.551800
15	0.077819	0.040404	1	-0.044021	0.282192	0.151436	0.522250
16	0.081900	0.040404	1	-0.187437	0.303248	0.122326	0.494150
17	0.080071	0.040404	1	-0.073973	0.285927	0.153610	0.541600
18	0.090821	0.040404	1	-0.084025	0.281929	0.166947	0.537600
19	0.088359	0.040404	1	-0.027479	0.341037	0.222604	0.560609
20	0.077617	0.040404	1	-0.150668	0.302536	0.127113	0.484750
21	0.066300	0.040404	1	-0.042923	0.293122	0.137318	0.482350
22	0.079337	0.040404	1	0.033403	0.304871	0.170097	0.508131
23	0.093359	0.040404	1	-0.117054	0.290007	0.158630	0.548600
24	0.100342	0.040404	1	-0.167034	0.283802	0.163843	0.541900
25	0.126120	0.040404	1	-0.101591	0.288617	0.154651	0.556100
26	0.060015	0.040404	1	-0.140411	0.297965	0.133885	0.481500
27	0.062302	0.040404	1	-0.072489	0.308086	0.167395	0.518693
28	0.113558	0.040404	1	-0.123897	0.290164	0.151891	0.569300
29	0.066757	0.040404	1	-0.018484	0.288898	0.130005	0.455900
30	0.101789	0.040404	1	-0.063554	0.301589	0.182452	0.510302
31	0.076619	0.040404	1	-0.098222	0.293166	0.146980	0.519000
32	0.107859	0.040404	1	-0.106868	0.294678	0.131831	0.497200
33	0.115115	0.040404	1	-0.081740	0.289357	0.163082	0.557300
34	0.063695	0.040404	1	-0.114716	0.299216	0.139103	0.488000
35	0.081613	0.040404	1	-0.105112	0.293663	0.130511	0.495554
36	0.074742	0.040404	1	-0.037031	0.282280	0.165974	0.516150
37	0.095581	0.040404	1	-0.111389	0.293133	0.159603	0.533350
38	0.064238	0.040404	1	-0.041580	0.316524	0.157485	0.493065
39	0.130658	0.040404	1	-0.127481	0.307836	0.130335	0.527850
40	0.094074	0.040404	1	-0.037239	0.280440	0.162964	0.565800
41	0.077803	0.040404	1	-0.109475	0.294000	0.138573	0.488450
42	0.068389	0.040404	1	0.039686	0.271824	0.151118	0.524203
43	0.059199	0.040404	1	-0.033388	0.291177	0.142291	0.497550
44	0.086587	0.040404	1	-0.028455	0.288329	0.135851	0.492350
45	0.073182	0.040404	1	-0.122930	0.296165	0.139501	0.505700
46	0.092966	0.040404	1	-0.067206	0.293338	0.151582	0.515250
47	0.089671	0.040404	1	-0.093033	0.336120	0.182003	0.510711
48	0.085799	0.040404	1	-0.081168	0.295756	0.143409	0.502300
49	0.106229	0.040404	1	-0.032055	0.292385	0.147220	0.524600
50	0.052850	0.040404	1	-0.024712	0.287109	0.144667	0.508800

(b) Power economy (continued)

	C	D	R	S	c_μ	c_σ	G
51	0.114463	0.040404	1	-0.050179	0.287927	0.132696	0.513200
52	0.096097	0.040404	1	-0.171549	0.301091	0.135622	0.504500
53	0.054559	0.040404	1	-0.050071	0.281362	0.155115	0.540850
54	0.057969	0.040404	1	-0.158163	0.294752	0.145961	0.508000
55	0.060298	0.040404	1	0.035572	0.284293	0.138264	0.498550
56	0.120463	0.040404	1	-0.162685	0.292661	0.159607	0.537250
57	0.079007	0.040404	1	-0.137168	0.310905	0.172098	0.539397
58	0.097859	0.040404	1	-0.070502	0.310628	0.171659	0.536080
59	0.075165	0.040404	1	0.013190	0.290442	0.140083	0.474900
60	0.100880	0.040404	1	-0.063428	0.286200	0.160913	0.539600
61	0.069155	0.040404	1	-0.026467	0.298512	0.116927	0.487050
62	0.084568	0.040404	1	-0.094879	0.292607	0.154308	0.503100
63	0.054151	0.040404	1	-0.032517	0.282704	0.154795	0.510800
64	0.106441	0.040404	1	-0.180393	0.283277	0.137606	0.482250
65	0.130094	0.040404	1	-0.025836	0.278055	0.153909	0.542600
66	0.121115	0.040404	1	-0.143829	0.286626	0.157534	0.544650
67	0.091387	0.040404	1	-0.154699	0.288587	0.157024	0.521000
68	0.071675	0.040404	1	-0.042227	0.293839	0.150715	0.508900
69	0.103209	0.040404	1	-0.175718	0.289109	0.157414	0.540250
70	0.103096	0.040404	1	-0.149077	0.286696	0.156196	0.523850
71	0.077618	0.040404	1	-0.083150	0.291563	0.140845	0.486550
72	0.139111	0.040404	1	-0.079405	0.292178	0.147390	0.544250
73	0.085304	0.040404	1	-0.061197	0.283941	0.133911	0.495950
74	0.124035	0.040404	1	-0.075489	0.283417	0.161558	0.501800
75	0.084683	0.040404	1	-0.092323	0.295781	0.124594	0.472750
76	0.134047	0.040404	1	-0.175208	0.289502	0.175459	0.554050
77	0.123629	0.040404	1	-0.108536	0.306113	0.121431	0.515400
78	0.088209	0.040404	1	-0.096905	0.289364	0.137163	0.512150
79	0.072689	0.040404	1	-0.041740	0.290739	0.140355	0.483950
80	0.083278	0.040404	1	-0.206074	0.290985	0.155096	0.526700
81	0.083913	0.040404	1	-0.140202	0.305537	0.170001	0.488643
82	0.085148	0.040404	1	-0.079432	0.309451	0.175654	0.532714
83	0.076238	0.040404	1	-0.182514	0.282377	0.158851	0.546300
84	0.076378	0.040404	1	-0.146801	0.289016	0.147888	0.491750
85	0.069792	0.040404	1	-0.002500	0.288503	0.131421	0.472400
86	0.083623	0.040404	1	-0.084103	0.310958	0.178885	0.537940
87	0.091103	0.040404	1	-0.015721	0.287810	0.131693	0.489700
88	0.087484	0.040404	1	0.010664	0.270908	0.169630	0.540000
89	0.066823	0.040404	1	-0.185615	0.299797	0.149544	0.528950
90	0.060647	0.040404	1	-0.071502	0.283748	0.166675	0.515700
91	0.115394	0.040404	1	-0.249781	0.296010	0.142122	0.498000
92	0.096718	0.040404	1	-0.086718	0.312597	0.179777	0.546884
93	0.093253	0.040404	1	-0.088740	0.295874	0.147422	0.515600
94	0.069575	0.040404	1	-0.069407	0.289295	0.153890	0.525000
95	0.069638	0.040404	1	-0.153946	0.314792	0.175466	0.524523
96	0.067895	0.040404	1	-0.068597	0.291962	0.136760	0.481800
97	0.064403	0.040404	1	-0.132907	0.298769	0.124608	0.446550
98	0.086748	0.040404	1	-0.062451	0.291162	0.145613	0.502350
99	0.089077	0.040404	1	-0.031101	0.288541	0.154386	0.548000
100	0.101126	0.040404	1	-0.032044	0.300393	0.118587	0.493100

(c) Gift economy

	C	D	R	S	c_μ	c_σ	G
1	0.046000	0.040404	1	-0.074410	0.293732	0.042905	0.244550
2	0.045762	0.040404	1	0.055772	0.291774	0.031149	0.214400
3	0.029174	0.040404	1	-0.143375	0.292206	0.067773	0.269000
4	0.015238	0.040404	1	-0.035811	0.313301	0.104751	0.267990
5	0.056349	0.040404	1	-0.032060	0.290688	0.069038	0.287100
6	0.041262	0.040404	1	-0.103265	0.293209	0.045204	0.270400
7	0.051691	0.040404	1	-0.026710	0.290589	0.061909	0.259650
8	0.028191	0.040404	1	0.017615	0.293533	0.043464	0.238250
9	0.075333	0.040404	1	0.123179	0.284501	0.048066	0.265200
10	0.038447	0.040404	1	-0.124786	0.289782	0.043931	0.244850
11	0.016040	0.040404	1	-0.053265	0.300843	0.033752	0.264150
12	0.062159	0.040404	1	-0.080939	0.295773	0.045166	0.265850
13	0.028714	0.040404	1	-0.036143	0.291323	0.031146	0.231600
14	0.040135	0.040404	1	-0.033871	0.296580	0.045141	0.269100
15	0.033349	0.040404	1	-0.031842	0.295597	0.036629	0.277450
16	0.020939	0.040404	1	0.063114	0.289881	0.064703	0.286150
17	0.045095	0.040404	1	-0.112304	0.292821	0.044583	0.256750
18	0.037389	0.040404	1	0.009368	0.293503	0.054136	0.267400
19	0.029048	0.040404	1	0.077057	0.286987	0.046025	0.249800
20	0.082667	0.040404	1	0.008346	0.288463	0.041496	0.227600
21	0.029149	0.040404	1	-0.103735	0.293236	0.054572	0.262500
22	0.025119	0.040404	1	-0.009466	0.294790	0.035312	0.267600
23	0.050119	0.040404	1	-0.017035	0.286236	0.052889	0.245850
24	0.043794	0.040404	1	-0.092508	0.289777	0.060400	0.268000
25	0.025056	0.040404	1	-0.053724	0.293494	0.037590	0.279450
26	0.033833	0.040404	1	0.101013	0.290659	0.048824	0.262152
27	0.042841	0.040404	1	-0.050373	0.290089	0.043436	0.242150
28	0.047126	0.040404	1	-0.031912	0.290915	0.056230	0.281400
29	0.040079	0.040404	1	-0.073935	0.292804	0.062573	0.275000
30	0.032191	0.040404	1	0.157887	0.286175	0.046704	0.243400
31	0.047323	0.040404	1	0.097343	0.291984	0.044808	0.243000
32	0.040389	0.040404	1	0.068001	0.296315	0.040921	0.283850
33	0.030595	0.040404	1	-0.083127	0.293093	0.041236	0.233650
34	0.039302	0.040404	1	0.043696	0.289558	0.038138	0.279800
35	0.025569	0.040404	1	-0.007922	0.297862	0.048398	0.286650
36	0.048992	0.040404	1	-0.106132	0.293097	0.032162	0.236350
37	0.032238	0.040404	1	-0.046447	0.291248	0.062340	0.280300
38	0.041571	0.040404	1	-0.164981	0.294744	0.042240	0.247750
39	0.025064	0.040404	1	0.029552	0.294635	0.052505	0.242550
40	0.031246	0.040404	1	0.108420	0.291541	0.050111	0.275450
41	0.023238	0.040404	1	-0.087411	0.294755	0.041743	0.230300
42	0.011571	0.040404	1	-0.043092	0.293065	0.033250	0.231950
43	0.018595	0.040404	1	0.129123	0.291162	0.044983	0.235700
44	0.044564	0.040404	1	0.009520	0.292819	0.048196	0.278050
45	0.039055	0.040404	1	-0.040895	0.297204	0.069814	0.289700
46	0.029119	0.040404	1	-0.107586	0.299710	0.046990	0.274500
47	0.059738	0.040404	1	-0.088115	0.291165	0.053353	0.273250
48	0.045675	0.040404	1	0.009732	0.290404	0.047866	0.278000
49	0.047778	0.040404	1	-0.120219	0.292235	0.043158	0.266800
50	0.019022	0.040404	1	0.058655	0.294691	0.057240	0.290500

(c) Gift economy (continued)

	C	D	R	S	c_μ	c_σ	G
51	0.039397	0.040404	1	0.053672	0.296795	0.072349	0.315600
52	0.037500	0.040404	1	0.107143	0.287362	0.068652	0.266300
53	0.036048	0.040404	1	-0.061080	0.291739	0.045989	0.262600
54	0.019619	0.040404	1	-0.068705	0.295081	0.034266	0.257502
55	0.036667	0.040404	1	-0.142635	0.297581	0.044501	0.266100
56	0.051286	0.040404	1	0.007874	0.295803	0.044146	0.267600
57	0.033095	0.040404	1	-0.042037	0.294414	0.043829	0.247400
58	0.077659	0.040404	1	-0.053415	0.288858	0.055203	0.285300
59	0.040848	0.040404	1	-0.098587	0.290661	0.061436	0.279450
60	0.046587	0.040404	1	0.115239	0.289056	0.044845	0.255150
61	0.038095	0.040404	1	-0.025685	0.293986	0.046667	0.287700
62	0.047728	0.040404	1	-0.000336	0.291996	0.046788	0.258650
63	0.050040	0.040404	1	-0.054121	0.295805	0.045748	0.277500
64	0.020024	0.040404	1	-0.019359	0.294242	0.054454	0.262200
65	0.032929	0.040404	1	0.014137	0.288767	0.047227	0.263750
66	0.051254	0.040404	1	-0.018050	0.289708	0.064001	0.293000
67	0.061635	0.040404	1	-0.047619	0.292628	0.029559	0.236250
68	0.053151	0.040404	1	-0.156823	0.297311	0.046796	0.300750
69	0.015857	0.040404	1	-0.022099	0.291459	0.043772	0.232150
70	0.045929	0.040404	1	0.130774	0.305228	0.124996	0.281658
71	0.048024	0.040404	1	-0.065862	0.292371	0.033922	0.268600
72	0.020968	0.040404	1	-0.059769	0.294170	0.062518	0.278650
73	0.035564	0.040404	1	-0.097220	0.288129	0.042947	0.224950
74	0.031595	0.040404	1	-0.020492	0.296337	0.062169	0.280850
75	0.026754	0.040404	1	0.011914	0.294022	0.040568	0.282600
76	0.022643	0.040404	1	0.002071	0.293041	0.054514	0.255350
77	0.036381	0.040404	1	-0.038050	0.292599	0.063407	0.275750
78	0.055683	0.040404	1	-0.048395	0.292911	0.033743	0.245250
79	0.030881	0.040404	1	0.005533	0.308409	0.112992	0.267286
80	0.068294	0.040404	1	-0.076434	0.295354	0.044244	0.262750
81	0.038691	0.040404	1	0.013376	0.289061	0.071503	0.289005
82	0.019516	0.040404	1	0.041626	0.299323	0.050673	0.314350
83	0.039000	0.040404	1	-0.104563	0.290765	0.043454	0.241600
84	0.058857	0.040404	1	0.017032	0.287527	0.042592	0.232650
85	0.031730	0.040404	1	0.080026	0.295067	0.058005	0.313700
86	0.041730	0.040404	1	-0.005825	0.291421	0.052553	0.254900
87	0.023825	0.040404	1	-0.055193	0.295950	0.053887	0.269850
88	0.029294	0.040404	1	-0.060395	0.292891	0.044903	0.255150
89	0.037487	0.040404	1	-0.047629	0.316888	0.108398	0.291558
90	0.028229	0.040404	1	0.025290	0.292062	0.064405	0.288900
91	0.039262	0.040404	1	-0.037975	0.294326	0.033440	0.249150
92	0.044714	0.040404	1	-0.051074	0.295490	0.045620	0.253601
93	0.057349	0.040404	1	0.040300	0.289278	0.046417	0.264600
94	0.049594	0.040404	1	0.035393	0.292916	0.037570	0.262550
95	0.022452	0.040404	1	-0.016540	0.295187	0.033054	0.244750
96	0.035476	0.040404	1	-0.030777	0.292612	0.055190	0.251250
97	0.038889	0.040404	1	0.045956	0.289605	0.065103	0.294050
98	0.049500	0.040404	1	-0.033990	0.293642	0.056713	0.287300
99	0.032071	0.040404	1	0.166606	0.287859	0.056194	0.270051
100	0.034929	0.040404	1	-0.076843	0.291572	0.061782	0.255900

(d) Concession economy

	C	D	R	S	c_μ	c_σ	G
1	0.04071	0.038485	0.036745	-0.074978	0.285831	0.052137	0.358648
2	0.04106	0.038081	0.005305	-0.045813	0.272890	0.056862	0.376629
3	0.05243	0.038182	0.021164	0.125173	0.282952	0.034380	0.396182
4	0.03037	0.038283	0.036939	0.001189	0.285101	0.048524	0.355725
5	0.02490	0.038182	0.047619	-0.012900	0.290627	0.037549	0.396782
6	0.02613	0.037879	0.037333	0.028483	0.276047	0.044884	0.441322
7	0.03120	0.037475	0.059299	-0.009758	0.286478	0.101904	0.365753
8	0.02466	0.038687	0.041776	0.009770	0.283550	0.056794	0.417801
9	0.02927	0.038788	0.026042	0.026020	0.291498	0.038696	0.345972
10	0.03555	0.038586	0.041885	-0.053559	0.294643	0.034883	0.358312
11	0.04519	0.038788	0.031250	0.021689	0.288130	0.050794	0.326987
12	0.03248	0.038081	0.053050	-0.040035	0.275023	0.061366	0.400115
13	0.052295	0.038485	0.036745	-0.021702	0.283796	0.061733	0.360055
14	0.042942	0.037879	0.016000	0.001699	0.282417	0.056271	0.373263
15	0.044063	0.038283	0.026385	-0.063811	0.286714	0.034221	0.319246
16	0.039300	0.038788	0.057292	-0.098588	0.290210	0.034612	0.336965
17	0.037890	0.039091	0.077519	0.055156	0.288853	0.049034	0.373126
18	0.036718	0.038283	0.042216	0.028449	0.272189	0.071605	0.407429
19	0.053671	0.038081	0.037135	0.027340	0.273293	0.067626	0.396708
20	0.038031	0.038788	0.026042	0.019337	0.283712	0.047205	0.423473
21	0.057382	0.038889	0.051948	0.067463	0.283024	0.053591	0.423681
22	0.047964	0.037879	0.053333	-0.096740	0.292217	0.089232	0.375276
23	0.035972	0.038788	0.046875	0.028959	0.280863	0.047378	0.457326
24	0.029732	0.038586	0.041885	-0.116920	0.287593	0.052884	0.358616
25	0.045243	0.037980	0.015957	0.011993	0.271178	0.067768	0.423096
26	0.038168	0.038283	0.073879	-0.034061	0.287151	0.045543	0.333482
27	0.035537	0.038687	0.031332	-0.019890	0.285005	0.054013	0.361182
28	0.039397	0.038485	0.036745	0.001712	0.292809	0.088791	0.357214
29	0.039784	0.038788	0.031250	0.034110	0.291084	0.090600	0.388613
30	0.033500	0.038485	0.026247	0.034605	0.288961	0.052743	0.378576
31	0.034402	0.038687	0.052219	0.063417	0.296345	0.035972	0.349922
32	0.023052	0.038283	0.036939	0.013318	0.271626	0.078440	0.421938
33	0.025222	0.038182	0.021164	0.054978	0.284019	0.060105	0.360198
34	0.039503	0.038485	0.026247	-0.055784	0.288114	0.053377	0.371549
35	0.032867	0.038384	0.010526	0.007132	0.280520	0.051739	0.392548
36	0.031020	0.038485	0.031496	0.036986	0.280265	0.050528	0.424847
37	0.039601	0.038485	0.041995	-0.063584	0.283925	0.051818	0.349921
38	0.030212	0.039091	0.031008	0.093926	0.288700	0.052742	0.428323
39	0.041191	0.038384	0.057895	-0.084955	0.282065	0.051717	0.339575
40	0.044368	0.038586	0.026178	0.024906	0.284692	0.046514	0.481132
41	0.025190	0.038182	0.052910	0.043254	0.280322	0.057324	0.405349
42	0.041786	0.038182	0.026455	-0.002470	0.275445	0.060210	0.370051
43	0.026218	0.039394	0.035897	-0.005945	0.291768	0.035468	0.358041
44	0.038520	0.038586	0.041885	0.024071	0.290252	0.038115	0.337901
45	0.016817	0.038687	0.041776	-0.023008	0.291023	0.044817	0.330116
46	0.031323	0.038182	0.037037	0.020555	0.279720	0.062189	0.394843
47	0.022628	0.038182	0.058201	-0.106016	0.293153	0.084505	0.370512
48	0.022873	0.038788	0.031250	-0.056024	0.294294	0.045423	0.331831
49	0.037254	0.038889	0.020779	0.038973	0.287847	0.053131	0.349199
50	0.046404	0.038687	0.031332	0.053804	0.283716	0.047014	0.401499

(d) Concession economy (continued)

	C	D	R	S	c_μ	c_σ	G
51	0.039201	0.038182	0.037037	-0.035249	0.286961	0.094459	0.398308
52	0.041362	0.038081	0.021220	0.092000	0.268619	0.082224	0.483843
53	0.021576	0.038687	0.036554	0.016880	0.284893	0.056360	0.404580
54	0.043255	0.038182	0.052910	0.023920	0.286205	0.098068	0.375510
55	0.050572	0.038788	0.031250	0.033322	0.281294	0.056462	0.420926
56	0.029437	0.038384	0.036842	-0.050926	0.281523	0.031291	0.348604
57	0.044752	0.038384	0.036842	-0.003444	0.304099	0.109825	0.397649
58	0.034924	0.038485	0.036745	0.064092	0.284410	0.047417	0.404309
59	0.036451	0.037475	0.048518	-0.000240	0.286900	0.050099	0.375030
60	0.033750	0.038081	0.026525	-0.012038	0.284811	0.043734	0.357440
61	0.027685	0.038485	0.041995	-0.027604	0.284012	0.042488	0.342511
62	0.026419	0.038687	0.052219	-0.044321	0.280501	0.061562	0.368074
63	0.039110	0.038586	0.052356	-0.158678	0.285148	0.059104	0.347726
64	0.040517	0.038384	0.036842	-0.096001	0.292802	0.031762	0.353267
65	0.035396	0.038990	0.056995	-0.056199	0.285777	0.056090	0.388367
66	0.044837	0.038283	0.031662	0.014688	0.292863	0.050774	0.381084
67	0.025266	0.038081	0.021220	-0.016517	0.278414	0.060792	0.397351
68	0.026038	0.038586	0.047120	-0.068895	0.285851	0.053876	0.380256
69	0.036093	0.038283	0.021108	-0.023040	0.288492	0.093718	0.364523
70	0.037377	0.038182	0.047619	0.005741	0.287479	0.056565	0.373849
71	0.025311	0.038283	0.052770	0.022040	0.280938	0.063256	0.378037
72	0.043926	0.038586	0.047120	-0.008354	0.283357	0.058658	0.352164
73	0.035834	0.038687	0.052219	-0.092034	0.281165	0.094173	0.425654
74	0.032052	0.039091	0.051680	-0.000989	0.281854	0.074305	0.415958
75	0.037857	0.038586	0.068063	-0.052970	0.295228	0.084132	0.339064
76	0.020338	0.038586	0.020942	0.079429	0.274075	0.049374	0.428240
77	0.041767	0.039091	0.025840	-0.057764	0.279478	0.054377	0.374876
78	0.031735	0.038586	0.041885	0.067418	0.277195	0.060499	0.430730
79	0.031230	0.038586	0.026178	-0.106456	0.289999	0.044471	0.380580
80	0.037437	0.037879	0.058667	-0.060195	0.285254	0.051097	0.379548
81	0.044281	0.037778	0.042781	-0.050388	0.283077	0.061136	0.365756
82	0.032421	0.038384	0.047368	-0.029043	0.285971	0.045144	0.392475
83	0.034060	0.038687	0.026110	0.013251	0.291624	0.089931	0.415933
84	0.044249	0.038788	0.020833	-0.009233	0.291647	0.033800	0.336755
85	0.030478	0.038384	0.042105	0.040978	0.297080	0.137865	0.413856
86	0.034433	0.038384	0.047368	0.005076	0.281700	0.056033	0.440599
87	0.044388	0.038485	0.062992	-0.025881	0.289633	0.088399	0.339776
88	0.046602	0.037778	0.021390	0.041032	0.284044	0.032763	0.388452
89	0.044173	0.038485	0.052493	-0.015896	0.286794	0.043789	0.350251
90	0.042732	0.037475	0.053908	0.047844	0.278843	0.042747	0.372179
91	0.049365	0.038586	0.036649	0.108381	0.284128	0.056851	0.343223
92	0.044896	0.038889	0.031169	-0.013341	0.292529	0.095033	0.411885
93	0.031352	0.037980	0.015957	0.063980	0.284449	0.047026	0.353483
94	0.052517	0.038283	0.015831	0.040187	0.275277	0.062685	0.407538
95	0.038323	0.037677	0.032172	-0.044258	0.286421	0.046447	0.318627
96	0.032517	0.039091	0.046512	0.007358	0.288315	0.037300	0.361330
97	0.042770	0.038687	0.026110	-0.002216	0.281473	0.046763	0.383649
98	0.041369	0.037475	0.043127	0.106256	0.270648	0.066873	0.378983
99	0.044010	0.038182	0.047619	-0.015969	0.286411	0.045623	0.314955
100	0.030129	0.038182	0.010582	0.008697	0.284882	0.104658	0.386309

(e) Watts–Strogatz model

	C	D	R	S	c_μ	c_σ	G
1	0.042762	0.040404	1	0.033691	0.308551	0.103763	0.228794
2	0.065238	0.040404	1	0.004667	0.285979	0.025037	0.169150
3	0.028119	0.040404	1	0.021341	0.291371	0.028681	0.201300
4	0.049762	0.040404	1	0.043213	0.287593	0.032760	0.227200
5	0.049167	0.040404	1	0.019464	0.289089	0.028915	0.193400
6	0.065405	0.040404	1	0.050194	0.287393	0.028774	0.212500
7	0.040373	0.040404	1	0.068323	0.291250	0.031128	0.206650
8	0.035111	0.040404	1	-0.072625	0.292040	0.042100	0.236000
9	0.042468	0.040404	1	-0.069640	0.293455	0.042958	0.217550
10	0.043167	0.040404	1	0.058843	0.289006	0.028377	0.189400
11	0.044976	0.040404	1	-0.058227	0.290901	0.028984	0.215250
12	0.030286	0.040404	1	0.091606	0.286794	0.042123	0.202300
13	0.038191	0.040404	1	0.006079	0.288983	0.042323	0.224850
14	0.046333	0.040404	1	0.001324	0.285780	0.028693	0.182000
15	0.029881	0.040404	1	-0.062872	0.294872	0.026490	0.195500
16	0.056952	0.040404	1	0.104489	0.283148	0.030590	0.196801
17	0.041000	0.040404	1	-0.017885	0.283533	0.026583	0.181900
18	0.032762	0.040404	1	-0.098521	0.295158	0.039767	0.207350
19	0.049476	0.040404	1	-0.139475	0.285878	0.039509	0.201150
20	0.029333	0.040404	1	-0.132915	0.287554	0.025265	0.170900
21	0.044476	0.040404	1	-0.046849	0.291807	0.028762	0.192350
22	0.044619	0.040404	1	-0.032996	0.289307	0.030099	0.204650
23	0.056571	0.040404	1	-0.083063	0.291408	0.049734	0.225100
24	0.022952	0.040404	1	-0.041131	0.294313	0.028869	0.193650
25	0.038619	0.040404	1	-0.037736	0.289147	0.027005	0.196200
26	0.018286	0.040404	1	-0.064420	0.291095	0.041218	0.210600
27	0.009952	0.040404	1	0.037780	0.290176	0.028421	0.190000
28	0.040365	0.040404	1	-0.106052	0.292033	0.050360	0.225100
29	0.060643	0.040404	1	-0.074996	0.289093	0.027322	0.200300
30	0.045714	0.040404	1	0.141990	0.282841	0.032136	0.209350
31	0.044857	0.040404	1	0.097848	0.287311	0.028422	0.189000
32	0.027095	0.040404	1	-0.018890	0.291297	0.044682	0.228650
33	0.017381	0.040404	1	-0.073993	0.289997	0.027425	0.194550
34	0.054214	0.040404	1	0.114973	0.288446	0.039485	0.195650
35	0.034691	0.040404	1	0.034351	0.288163	0.029653	0.191000
36	0.045762	0.040404	1	-0.017682	0.289978	0.042198	0.207650
37	0.064952	0.040404	1	0.108341	0.290318	0.043811	0.226150
38	0.028905	0.040404	1	0.090142	0.290406	0.026695	0.186400
39	0.038262	0.040404	1	0.115578	0.288495	0.028751	0.194000
40	0.024333	0.040404	1	-0.055024	0.288441	0.025025	0.188950
41	0.059524	0.040404	1	0.099200	0.287933	0.029729	0.185600
42	0.038810	0.040404	1	0.019179	0.292867	0.033436	0.227550
43	0.038643	0.040404	1	0.037990	0.287314	0.041915	0.209350
44	0.063595	0.040404	1	-0.228265	0.285248	0.024092	0.214050
45	0.036619	0.040404	1	-0.246796	0.281122	0.023580	0.181100
46	0.053571	0.040404	1	0.008658	0.287256	0.040628	0.212400
47	0.020238	0.040404	1	-0.079976	0.292122	0.041091	0.212400
48	0.055048	0.040404	1	-0.116408	0.285312	0.037755	0.189350
49	0.016167	0.040404	1	0.109395	0.287264	0.031287	0.191000
50	0.069254	0.040404	1	-0.191170	0.293707	0.039449	0.214650

(e) Watts–Strogatz model (continued)

	C	D	R	S	c_μ	c_σ	G
51	0.041286	0.040404	1	-0.024480	0.288448	0.030560	0.198250
52	0.037619	0.040404	1	0.124397	0.289240	0.027276	0.173350
53	0.039754	0.040404	1	-0.099691	0.311028	0.102276	0.229246
54	0.038952	0.040404	1	0.007418	0.293211	0.030792	0.214700
55	0.052310	0.040404	1	0.081684	0.289720	0.029522	0.203800
56	0.065429	0.040404	1	-0.006127	0.284872	0.027827	0.188200
57	0.040619	0.040404	1	-0.045260	0.288189	0.027705	0.202050
58	0.041373	0.040404	1	0.024620	0.290484	0.029421	0.210800
59	0.039810	0.040404	1	-0.082875	0.288468	0.024231	0.183600
60	0.055976	0.040404	1	-0.012384	0.291970	0.043788	0.247700
61	0.066905	0.040404	1	-0.065699	0.285530	0.026444	0.191150
62	0.027921	0.040404	1	-0.035115	0.292645	0.028242	0.193900
63	0.022476	0.040404	1	0.050688	0.287391	0.028907	0.190500
64	0.051119	0.040404	1	-0.040208	0.286700	0.024543	0.184300
65	0.048619	0.040404	1	0.053755	0.286607	0.027348	0.180400
66	0.045500	0.040404	1	-0.054782	0.291600	0.030026	0.197850
67	0.033714	0.040404	1	-0.098766	0.289800	0.025303	0.195900
68	0.052691	0.040404	1	0.038293	0.289621	0.050901	0.230400
69	0.044571	0.040404	1	-0.023754	0.287823	0.030661	0.217450
70	0.033318	0.040404	1	-0.160214	0.292506	0.026542	0.195400
71	0.036151	0.040404	1	-0.155993	0.294863	0.027572	0.229750
72	0.038238	0.040404	1	-0.047434	0.290296	0.028768	0.197100
73	0.023429	0.040404	1	-0.080945	0.289349	0.022506	0.179050
74	0.051286	0.040404	1	0.046366	0.286349	0.050942	0.222000
75	0.046762	0.040404	1	-0.053901	0.293251	0.052380	0.261100
76	0.037508	0.040404	1	-0.077903	0.287823	0.025817	0.192250
77	0.007667	0.040404	1	-0.065520	0.296423	0.027275	0.214300
78	0.089905	0.040404	1	-0.081455	0.286739	0.039910	0.203100
79	0.048373	0.040404	1	-0.080831	0.289098	0.025770	0.204750
80	0.073310	0.040404	1	-0.018011	0.283867	0.029371	0.209050
81	0.032286	0.040404	1	0.045229	0.291809	0.027979	0.189600
82	0.051429	0.040404	1	-0.059128	0.292131	0.040065	0.211300
83	0.046849	0.040404	1	0.086569	0.292113	0.031750	0.209950
84	0.036238	0.040404	1	-0.115124	0.288769	0.026941	0.204650
85	0.058691	0.040404	1	-0.118633	0.285861	0.048161	0.229350
86	0.045857	0.040404	1	0.052105	0.286126	0.028443	0.187850
87	0.076691	0.040404	1	-0.138892	0.289461	0.025498	0.197950
88	0.031667	0.040404	1	-0.080553	0.291260	0.042465	0.218900
89	0.028762	0.040404	1	0.010676	0.286402	0.030165	0.204400
90	0.058048	0.040404	1	-0.026368	0.288146	0.028826	0.212000
91	0.038167	0.040404	1	-0.116344	0.287612	0.027300	0.193400
92	0.049524	0.040404	1	-0.104972	0.289621	0.026250	0.200350
93	0.042976	0.040404	1	0.023222	0.290667	0.039990	0.200800
94	0.087619	0.040404	1	-0.045725	0.285281	0.042054	0.217618
95	0.057238	0.040404	1	-0.060041	0.290843	0.058465	0.230300
96	0.039119	0.040404	1	-0.099859	0.291097	0.029396	0.208400
97	0.034191	0.040404	1	0.035302	0.287819	0.025916	0.197050
98	0.043286	0.040404	1	-0.136284	0.288276	0.040935	0.213850
99	0.047786	0.040404	1	-0.097368	0.288741	0.025194	0.193400
100	0.050905	0.040404	1	-0.087140	0.288510	0.038745	0.197700

(f) Watts–Strogatz derivative model

	C	D	R	S	c_μ	c_σ	G
1	0.083603	0.039495	0.312020	0.188565	0.283320	0.104680	0.418817
2	0.085853	0.039293	0.323907	0.226872	0.284329	0.059025	0.388262
3	0.082702	0.039495	0.327366	0.075197	0.282422	0.066400	0.442068
4	0.079576	0.038889	0.327273	0.175565	0.281731	0.063179	0.389885
5	0.097852	0.039495	0.312020	0.102882	0.269615	0.083518	0.434840
6	0.071102	0.038788	0.307292	0.182192	0.281203	0.106015	0.470526
7	0.073334	0.039394	0.302564	0.152113	0.270558	0.077918	0.460327
8	0.088464	0.039697	0.305344	0.091044	0.285479	0.099527	0.417928
9	0.080299	0.039596	0.290816	0.132949	0.286532	0.105433	0.413691
10	0.075748	0.039596	0.295918	0.101975	0.285033	0.107864	0.425860
11	0.093049	0.039596	0.331633	0.046905	0.285896	0.101943	0.453828
12	0.079440	0.038485	0.335958	0.096343	0.282542	0.095208	0.467923
13	0.077711	0.039192	0.319588	0.189499	0.278413	0.075025	0.397783
14	0.077741	0.039091	0.320413	0.187837	0.279580	0.102469	0.458123
15	0.085339	0.038990	0.316062	0.158651	0.271057	0.062187	0.452377
16	0.086784	0.039091	0.315245	0.122943	0.272890	0.062517	0.428114
17	0.089485	0.039192	0.314433	0.156800	0.280644	0.058334	0.389563
18	0.088673	0.038889	0.311688	0.086803	0.286851	0.090508	0.427346
19	0.070460	0.038990	0.316062	0.201016	0.282818	0.061692	0.438258
20	0.088756	0.039697	0.305344	0.214414	0.276251	0.074467	0.440746
21	0.099952	0.038687	0.318538	0.243092	0.283572	0.105123	0.430571
22	0.075736	0.038990	0.305699	0.133050	0.278594	0.108933	0.448818
23	0.076082	0.039798	0.314721	0.022674	0.292232	0.091389	0.374597
24	0.080141	0.039394	0.328205	0.171956	0.281426	0.080125	0.445430
25	0.097805	0.039495	0.306905	0.094858	0.276497	0.112206	0.481826
26	0.085941	0.039192	0.319588	0.140677	0.278635	0.103460	0.411259
27	0.078098	0.039697	0.310433	0.147681	0.283919	0.068888	0.426183
28	0.091161	0.038889	0.322078	0.159210	0.285140	0.105161	0.430159
29	0.072823	0.039697	0.295165	0.177590	0.286554	0.113621	0.449535
30	0.087200	0.039394	0.292308	0.143150	0.292985	0.091724	0.426746
31	0.091025	0.039697	0.310433	0.186446	0.283900	0.105395	0.451365
32	0.075492	0.039596	0.336735	0.112633	0.289352	0.095022	0.449725
33	0.102360	0.039596	0.295918	0.234331	0.293930	0.123304	0.414510
34	0.085623	0.039394	0.307692	0.209117	0.279971	0.071863	0.434343
35	0.084161	0.038889	0.332468	0.114840	0.286754	0.063564	0.408309
36	0.081726	0.039091	0.320413	0.178330	0.283926	0.104161	0.394576
37	0.093370	0.038990	0.336788	0.167518	0.293694	0.129253	0.464775
38	0.075272	0.038990	0.316062	0.198851	0.283746	0.055806	0.412872
39	0.073176	0.038788	0.322917	0.216401	0.281563	0.103812	0.456167
40	0.102395	0.039596	0.316327	0.214487	0.277126	0.060005	0.473982
41	0.066278	0.039192	0.319588	0.157362	0.295508	0.150230	0.411738
42	0.074961	0.039293	0.313625	0.094400	0.283234	0.102937	0.436072
43	0.073703	0.038788	0.317708	0.130078	0.277696	0.094432	0.419723
44	0.086746	0.039899	0.313924	0.213265	0.294193	0.128136	0.445729
45	0.081881	0.039596	0.321429	0.169915	0.274204	0.071219	0.488530
46	0.086198	0.038990	0.316062	0.245978	0.273574	0.068742	0.434555
47	0.077111	0.039091	0.310078	0.187650	0.278297	0.109539	0.471726
48	0.083865	0.039697	0.300254	0.057985	0.275125	0.079342	0.412210
49	0.065595	0.038990	0.300518	0.099781	0.279550	0.069759	0.435430
50	0.085141	0.038889	0.311688	0.206819	0.271641	0.091388	0.447471

(f) Watts–Strogatz derivative model (continued)

	C	D	R	S	c_{μ}	c_{σ}	G
51	0.086385	0.038687	0.323760	0.153823	0.279834	0.107079	0.445700
52	0.069522	0.039394	0.317949	0.150188	0.293012	0.063274	0.427689
53	0.080461	0.040000	0.323232	0.184017	0.302096	0.145077	0.457875
54	0.092398	0.039091	0.304910	0.037769	0.274317	0.059580	0.353682
55	0.080508	0.039091	0.315245	0.246540	0.281566	0.062194	0.459578
56	0.080870	0.039192	0.304124	0.167062	0.279755	0.058354	0.435474
57	0.069407	0.039495	0.301790	0.186624	0.271836	0.077588	0.423339
58	0.078436	0.039495	0.301790	0.096987	0.279280	0.070228	0.400321
59	0.070579	0.039192	0.304124	0.192324	0.278228	0.060234	0.365050
60	0.068290	0.038889	0.296104	0.078756	0.274291	0.070322	0.409409
61	0.077350	0.039495	0.301790	0.093605	0.278570	0.070834	0.430426
62	0.071518	0.039697	0.300254	0.203002	0.285867	0.135529	0.541080
63	0.092278	0.038586	0.319372	0.157687	0.262408	0.091443	0.457892
64	0.085243	0.039394	0.333333	0.181003	0.285368	0.103098	0.386282
65	0.072131	0.038788	0.328125	0.249584	0.274668	0.109820	0.455636
66	0.103919	0.039293	0.313625	0.180360	0.271718	0.084892	0.436436
67	0.086491	0.039798	0.294416	0.145896	0.278561	0.073927	0.420142
68	0.103403	0.039495	0.317136	0.117841	0.280346	0.062849	0.425341
69	0.071376	0.038889	0.322078	0.241450	0.274539	0.114711	0.457381
70	0.072787	0.039495	0.301790	0.135263	0.278065	0.080844	0.411848
71	0.084233	0.039293	0.329049	0.192997	0.299606	0.121566	0.443833
72	0.072954	0.040101	0.322418	0.066658	0.280240	0.075145	0.417230
73	0.103862	0.039091	0.310078	0.126254	0.270125	0.074987	0.427557
74	0.076986	0.039293	0.323907	0.076180	0.275042	0.074300	0.431387
75	0.077890	0.039394	0.302564	0.183199	0.278056	0.057749	0.424053
76	0.092759	0.038687	0.308094	0.108412	0.277935	0.084862	0.421678
77	0.091931	0.039091	0.304910	0.119842	0.280421	0.084309	0.453198
78	0.098642	0.039495	0.332481	0.114517	0.282209	0.063499	0.393568
79	0.077566	0.039091	0.330749	0.063815	0.274812	0.069002	0.465772
80	0.106957	0.039394	0.323077	0.129676	0.285353	0.075021	0.480617
81	0.087330	0.039495	0.306905	0.204099	0.285184	0.096432	0.389607
82	0.066115	0.039293	0.308483	0.143410	0.287927	0.102297	0.438148
83	0.095258	0.039697	0.305344	0.114882	0.274505	0.069432	0.386087
84	0.100272	0.039697	0.300254	0.131091	0.299358	0.119119	0.379859
85	0.075657	0.039495	0.317136	0.138212	0.271505	0.085107	0.456101
86	0.096096	0.038889	0.311688	0.167809	0.281655	0.067060	0.481415
87	0.109527	0.039091	0.315245	0.044140	0.277182	0.098207	0.413869
88	0.082102	0.039495	0.317136	0.126349	0.286926	0.078722	0.405987
89	0.090003	0.039192	0.314433	0.095560	0.261938	0.084619	0.412052
90	0.091111	0.039091	0.304910	0.063716	0.284713	0.070418	0.496267
91	0.078106	0.039091	0.315245	0.140130	0.285920	0.099292	0.406225
92	0.084823	0.038788	0.312500	0.144434	0.278386	0.077587	0.457492
93	0.088677	0.039394	0.302564	0.097880	0.293873	0.105179	0.374570
94	0.089186	0.039798	0.299492	0.175867	0.281568	0.080283	0.452497
95	0.076724	0.039091	0.315245	0.165958	0.292653	0.093743	0.460336
96	0.080021	0.039495	0.296675	0.103484	0.271580	0.078211	0.406729
97	0.075641	0.039495	0.337596	0.200594	0.286216	0.117505	0.488775
98	0.098712	0.038384	0.310526	0.166042	0.262101	0.117669	0.463747
99	0.100108	0.039596	0.311224	0.152898	0.282707	0.065653	0.425403
100	0.079519	0.039495	0.322251	0.221819	0.287027	0.065479	0.388478

(g) Barabási–Albert model

	C	D	R	S	c_μ	c_σ	G
1	0.038212	0.040404	1	-0.138138	0.297546	0.120085	0.467600
2	0.074780	0.040404	1	-0.001886	0.306864	0.177164	0.520251
3	0.087852	0.040404	1	-0.080188	0.284857	0.147176	0.502750
4	0.079505	0.040404	1	0.015052	0.288874	0.140497	0.496200
5	0.101406	0.040404	1	0.109245	0.296221	0.174381	0.495528
6	0.082344	0.040404	1	-0.060485	0.292338	0.147076	0.531850
7	0.081536	0.040404	1	-0.125915	0.293392	0.132723	0.450150
8	0.067003	0.040404	1	-0.202303	0.293516	0.136723	0.482450
9	0.114559	0.040404	1	-0.023651	0.307307	0.159900	0.496314
10	0.105073	0.040404	1	-0.040801	0.286113	0.135270	0.493850
11	0.053253	0.040404	1	0.008349	0.306470	0.158132	0.452915
12	0.066874	0.040404	1	-0.025904	0.295673	0.124921	0.479150
13	0.095690	0.040404	1	-0.133592	0.286197	0.152076	0.520600
14	0.108037	0.040404	1	-0.106436	0.309086	0.178888	0.534623
15	0.085178	0.040404	1	-0.100017	0.297547	0.136102	0.515650
16	0.107644	0.040404	1	-0.071815	0.307885	0.177050	0.520603
17	0.073142	0.040404	1	-0.077235	0.289114	0.136439	0.499000
18	0.065366	0.040404	1	-0.068147	0.310662	0.159132	0.479347
19	0.079206	0.040404	1	-0.028353	0.296943	0.125898	0.467450
20	0.093138	0.040404	1	-0.121647	0.295303	0.129207	0.487850
21	0.095479	0.040404	1	-0.081473	0.288590	0.145934	0.498400
22	0.063281	0.040404	1	-0.084673	0.296317	0.134876	0.493650
23	0.087158	0.040404	1	-0.157709	0.291029	0.137281	0.498150
24	0.055191	0.040404	1	-0.113524	0.294929	0.142903	0.499500
25	0.064169	0.040404	1	-0.062588	0.292565	0.113281	0.433700
26	0.086595	0.040404	1	-0.073484	0.289481	0.162900	0.544450
27	0.086295	0.040404	1	-0.069069	0.299992	0.106541	0.457200
28	0.063721	0.040404	1	-0.057390	0.285524	0.147151	0.500400
29	0.086846	0.040404	1	0.066009	0.300755	0.175688	0.513467
30	0.079261	0.040404	1	0.019074	0.283542	0.118985	0.474861
31	0.074472	0.040404	1	-0.007774	0.291058	0.141962	0.492750
32	0.075053	0.040404	1	-0.038059	0.287852	0.139385	0.474850
33	0.088322	0.040404	1	-0.176576	0.292449	0.140369	0.492800
34	0.084463	0.040404	1	-0.120789	0.292211	0.137367	0.500800
35	0.041076	0.040404	1	-0.076790	0.298000	0.105432	0.423550
36	0.077223	0.040404	1	-0.158936	0.294740	0.144215	0.527251
37	0.090904	0.040404	1	-0.168388	0.298813	0.138979	0.486750
38	0.088019	0.040404	1	-0.042222	0.309512	0.167402	0.510151
39	0.061949	0.040404	1	-0.079132	0.313340	0.168191	0.509246
40	0.094472	0.040404	1	-0.007725	0.288027	0.140422	0.503200
41	0.051529	0.040404	1	-0.069068	0.305828	0.187314	0.517437
42	0.058962	0.040404	1	-0.029952	0.283119	0.155206	0.517100
43	0.088521	0.040404	1	-0.068364	0.313385	0.160351	0.500302
44	0.057434	0.040404	1	0.086603	0.342672	0.209437	0.462335
45	0.067807	0.040404	1	-0.105558	0.296499	0.133623	0.479550
46	0.049837	0.040404	1	-0.081628	0.292630	0.131827	0.451950
47	0.056599	0.040404	1	-0.233314	0.297081	0.123779	0.461750
48	0.057496	0.040404	1	-0.157648	0.309381	0.188710	0.539548
49	0.067413	0.040404	1	-0.063973	0.291684	0.150619	0.513300
50	0.098393	0.040404	1	-0.099774	0.289843	0.130253	0.440950

(g) Barabási–Albert model (continued)

	C	D	R	S	c_{μ}	c_{σ}	G
51	0.088309	0.040404	1	-0.066630	0.294708	0.135247	0.504800
52	0.103757	0.040404	1	-0.127100	0.289958	0.153034	0.501950
53	0.115923	0.040404	1	-0.150913	0.289801	0.147906	0.484450
54	0.061385	0.040404	1	-0.070865	0.294697	0.138371	0.490450
55	0.061191	0.040404	1	-0.062759	0.300360	0.116377	0.466400
56	0.077764	0.040404	1	-0.124096	0.291791	0.149593	0.493300
57	0.097770	0.040404	1	-0.127187	0.289376	0.153479	0.511700
58	0.064410	0.040404	1	-0.220894	0.303333	0.125936	0.467300
59	0.098480	0.040404	1	-0.176866	0.289811	0.154134	0.526700
60	0.065165	0.040404	1	-0.079598	0.294721	0.133529	0.475000
61	0.075403	0.040404	1	-0.124041	0.317963	0.173109	0.511156
62	0.050918	0.040404	1	-0.020712	0.292617	0.118945	0.457250
63	0.090225	0.040404	1	-0.033506	0.287016	0.148196	0.514150
64	0.085337	0.040404	1	-0.080346	0.288566	0.108182	0.430800
65	0.059339	0.040404	1	-0.052692	0.311658	0.169902	0.478040
66	0.067563	0.040404	1	-0.057756	0.312544	0.163932	0.489497
67	0.074181	0.040404	1	-0.094579	0.296396	0.130122	0.481700
68	0.090591	0.040404	1	0.012328	0.282356	0.150598	0.505900
69	0.056086	0.040404	1	0.043249	0.284630	0.116868	0.439650
70	0.075797	0.040404	1	-0.043226	0.286457	0.118530	0.463550
71	0.080454	0.040404	1	-0.067515	0.292577	0.124580	0.486850
72	0.076334	0.040404	1	-0.000197	0.296091	0.125446	0.487150
73	0.076941	0.040404	1	-0.163351	0.303515	0.132539	0.493800
74	0.070947	0.040404	1	-0.062209	0.305951	0.165957	0.476432
75	0.053518	0.040404	1	-0.022524	0.290739	0.122974	0.453350
76	0.068282	0.040404	1	-0.079293	0.291822	0.145093	0.498800
77	0.044644	0.040404	1	0.009359	0.287404	0.139345	0.485850
78	0.058154	0.040404	1	-0.056856	0.293930	0.142690	0.491150
79	0.097811	0.040404	1	-0.037718	0.286181	0.150836	0.490700
80	0.070042	0.040404	1	-0.033620	0.308749	0.168848	0.476382
81	0.065355	0.040404	1	-0.001474	0.292682	0.137378	0.491850
82	0.073358	0.040404	1	-0.011178	0.292504	0.128915	0.479050
83	0.080624	0.040404	1	-0.086817	0.286966	0.155594	0.514750
84	0.116973	0.040404	1	-0.124618	0.290023	0.167571	0.560950
85	0.089243	0.040404	1	-0.045198	0.283864	0.147316	0.519950
86	0.051922	0.040404	1	-0.136226	0.294541	0.123163	0.461300
87	0.090612	0.040404	1	-0.120643	0.299697	0.110899	0.448250
88	0.052337	0.040404	1	-0.032787	0.292973	0.136869	0.475400
89	0.069884	0.040404	1	-0.140449	0.276204	0.175134	0.543300
90	0.070740	0.040404	1	-0.116230	0.280154	0.156994	0.497150
91	0.056341	0.040404	1	-0.086302	0.293076	0.137335	0.486550
92	0.058945	0.040404	1	-0.092884	0.292573	0.128057	0.479000
93	0.084684	0.040404	1	-0.050077	0.293299	0.137146	0.477100
94	0.077382	0.040404	1	-0.134646	0.297411	0.140394	0.511250
95	0.084350	0.040404	1	-0.006117	0.297028	0.178405	0.512663
96	0.082752	0.040404	1	-0.124360	0.303097	0.127244	0.510950
97	0.059895	0.040404	1	-0.072431	0.311948	0.145329	0.469699
98	0.072804	0.040404	1	-0.067698	0.302408	0.133761	0.504800
99	0.094186	0.040404	1	-0.284663	0.314497	0.174183	0.499698
100	0.065449	0.040404	1	-0.003379	0.289088	0.149226	0.518800

(h) Watts–Strogatz model

	ρ								
	0	0.01	0.05	0.1	0.2	0.4	0.7	0.85	1
1	0.497938	0.369310	0.077606	0.149238	0.097667	0.162150	0.228794	0.238700	0.225250
2	0.497938	0.495288	0.140896	0.097518	0.112298	0.157550	0.169150	0.232200	0.232100
3	0.497938	0.350663	0.132280	0.081031	0.119663	0.173051	0.201300	0.221550	0.241250
4	0.497938	0.439477	0.067985	0.066146	0.119204	0.175656	0.227200	0.221550	0.245750
5	0.497938	0.214457	0.061154	0.156786	0.093597	0.154752	0.193400	0.212650	0.241100
6	0.497938	0.386622	0.115321	0.079025	0.105785	0.153800	0.212500	0.248844	0.225900
7	0.497938	0.247360	0.077079	0.086944	0.105151	0.168500	0.206650	0.195400	0.264800
8	0.497938	0.408189	0.121473	0.067826	0.117715	0.161150	0.236000	0.238500	0.258500
9	0.497938	0.261142	0.077232	0.139231	0.096681	0.177900	0.217550	0.203500	0.230800
10	0.497938	0.496782	0.139076	0.079693	0.117683	0.144600	0.189400	0.235500	0.247850
11	0.497938	0.397568	0.215803	0.070533	0.123112	0.149600	0.215250	0.242700	0.255100
12	0.497938	0.438348	0.286257	0.085181	0.110002	0.159150	0.202300	0.246450	0.240250
13	0.497938	0.482341	0.248075	0.077294	0.110811	0.178250	0.224850	0.220350	0.224350
14	0.497938	0.303780	0.154448	0.103835	0.104320	0.174350	0.182000	0.197350	0.229750
15	0.497938	0.387432	0.081182	0.102359	0.093714	0.155051	0.195500	0.229800	0.211600
16	0.497938	0.335865	0.093568	0.080863	0.110351	0.162854	0.196801	0.220400	0.243900
17	0.497938	0.475038	0.094353	0.176358	0.122786	0.162507	0.181900	0.212050	0.232802
18	0.497938	0.348721	0.096373	0.084312	0.130567	0.171350	0.207350	0.223650	0.241800
19	0.497938	0.495339	0.148584	0.078654	0.082010	0.129251	0.201150	0.222700	0.227050
20	0.497938	0.480551	0.151973	0.088045	0.119060	0.199950	0.170900	0.244350	0.228000
21	0.497938	0.496738	0.164212	0.126443	0.107987	0.173400	0.192350	0.221450	0.242950
22	0.497938	0.476132	0.078749	0.093718	0.117572	0.150401	0.204650	0.228450	0.233300
23	0.497938	0.497786	0.064545	0.088955	0.139961	0.190051	0.225100	0.246100	0.263250
24	0.497938	0.259812	0.213201	0.072525	0.110688	0.166350	0.193650	0.231800	0.243150
25	0.497938	0.497938	0.136394	0.106926	0.121406	0.144650	0.196200	0.221400	0.223650
26	0.497938	0.497938	0.261218	0.080075	0.111209	0.165950	0.210600	0.226900	0.226600
27	0.497938	0.497938	0.092054	0.086964	0.112888	0.135350	0.190000	0.224850	0.264950
28	0.497938	0.487319	0.099703	0.082936	0.100276	0.169900	0.225100	0.202450	0.234150
29	0.497938	0.240976	0.123282	0.075826	0.089396	0.169500	0.200300	0.220350	0.266050
30	0.497938	0.450335	0.116788	0.084381	0.118212	0.172802	0.209350	0.222300	0.259350
31	0.497938	0.475828	0.116307	0.087808	0.100541	0.162900	0.189000	0.225800	0.239850
32	0.497938	0.460296	0.295171	0.108224	0.116312	0.167300	0.228650	0.235300	0.251500
33	0.497938	0.497938	0.175041	0.080587	0.106952	0.152004	0.194550	0.250150	0.246300
34	0.497938	0.492536	0.128431	0.197697	0.135664	0.169300	0.195650	0.211550	0.235150
35	0.497938	0.298412	0.079854	0.095766	0.105219	0.163351	0.191000	0.239100	0.250350
36	0.497938	0.219752	0.068188	0.084555	0.116107	0.169152	0.207650	0.238000	0.242100
37	0.497938	0.497938	0.128113	0.084783	0.083349	0.169250	0.226150	0.245200	0.223700
38	0.497938	0.406100	0.172560	0.093683	0.114304	0.179850	0.186400	0.216050	0.251700
39	0.497938	0.456129	0.061164	0.102022	0.109018	0.147357	0.194000	0.247100	0.223350
40	0.497938	0.396362	0.083989	0.086049	0.108453	0.133052	0.188950	0.212000	0.259050
41	0.497938	0.288960	0.078728	0.067402	0.119118	0.153751	0.185600	0.240151	0.245200
42	0.497938	0.497938	0.167555	0.068470	0.114389	0.142600	0.227550	0.239950	0.218700
43	0.497938	0.318014	0.147697	0.137778	0.125758	0.155051	0.209350	0.218750	0.234700
44	0.497938	0.272079	0.126075	0.083127	0.131910	0.167350	0.214050	0.223200	0.239950
45	0.497938	0.334713	0.267708	0.078776	0.117362	0.161600	0.181100	0.202000	0.250000
46	0.497938	0.496300	0.232206	0.077737	0.103103	0.140050	0.212400	0.223050	0.217150
47	0.497938	0.330817	0.105015	0.088376	0.111581	0.159301	0.212400	0.225650	0.244950
48	0.497938	0.336382	0.110065	0.075230	0.113154	0.169000	0.189350	0.213550	0.205200
49	0.497938	0.207297	0.095057	0.087922	0.094177	0.155303	0.191000	0.184650	0.242050
50	0.497938	0.343585	0.073537	0.091965	0.113613	0.179350	0.214650	0.223350	0.250750

(h) Watts–Strogatz model (continued)

	ρ								
	0	0.01	0.05	0.1	0.2	0.4	0.7	0.85	1
51	0.497938	0.437647	0.089151	0.076420	0.118036	0.130202	0.198250	0.196200	0.222500
52	0.497938	0.499550	0.065537	0.097776	0.111168	0.195750	0.173350	0.212750	0.229350
53	0.497938	0.485495	0.115219	0.182420	0.125850	0.123602	0.229246	0.223920	0.235900
54	0.497938	0.461990	0.270857	0.072868	0.124120	0.183200	0.214700	0.231600	0.258844
55	0.497938	0.443322	0.085784	0.074874	0.107949	0.162652	0.203800	0.190550	0.229200
56	0.497938	0.420360	0.090279	0.179954	0.103630	0.186600	0.188200	0.204850	0.245100
57	0.497938	0.457816	0.088708	0.090741	0.111394	0.164700	0.202050	0.237300	0.226000
58	0.497938	0.455610	0.131679	0.089444	0.115627	0.190050	0.210800	0.238800	0.248150
59	0.497938	0.497938	0.233805	0.096502	0.104115	0.166750	0.183600	0.242800	0.224500
60	0.497938	0.288147	0.088169	0.155760	0.104965	0.159700	0.247700	0.241600	0.224925
61	0.497938	0.438287	0.076817	0.085419	0.114046	0.165300	0.191150	0.196300	0.261300
62	0.497938	0.333993	0.085481	0.086092	0.106223	0.133350	0.193900	0.216650	0.227800
63	0.497938	0.288780	0.148591	0.172773	0.100228	0.170251	0.190500	0.209700	0.214900
64	0.497938	0.243595	0.163881	0.080492	0.113123	0.162150	0.184300	0.225450	0.226500
65	0.497938	0.497771	0.070472	0.121680	0.119966	0.172100	0.180400	0.209300	0.246633
66	0.497938	0.198862	0.085565	0.097383	0.107509	0.179851	0.197850	0.225150	0.266550
67	0.497938	0.483172	0.066488	0.086974	0.107148	0.153918	0.195900	0.209050	0.228600
68	0.497938	0.498339	0.209561	0.086867	0.109274	0.160515	0.230400	0.214200	0.202700
69	0.497938	0.289500	0.128428	0.144958	0.092028	0.134811	0.217450	0.191850	0.216800
70	0.497938	0.365613	0.382837	0.090374	0.113826	0.173500	0.195400	0.186700	0.233750
71	0.497938	0.447445	0.194124	0.145280	0.100912	0.177800	0.229750	0.226600	0.223450
72	0.497938	0.493717	0.086403	0.105622	0.135936	0.170503	0.197100	0.233000	0.232900
73	0.497938	0.271375	0.253515	0.094753	0.096137	0.163601	0.179050	0.232150	0.212600
74	0.497938	0.487809	0.276353	0.091934	0.113022	0.176300	0.222000	0.225700	0.217100
75	0.497938	0.212736	0.090050	0.077217	0.111565	0.170700	0.261100	0.234300	0.238350
76	0.497938	0.497750	0.251710	0.083578	0.112473	0.150551	0.192250	0.231050	0.234700
77	0.497938	0.492861	0.046240	0.082083	0.120773	0.166400	0.214300	0.209550	0.251151
78	0.497938	0.301743	0.102568	0.088809	0.139624	0.137002	0.203100	0.248750	0.234250
79	0.497938	0.300710	0.228749	0.136756	0.138861	0.163350	0.204750	0.218200	0.231200
80	0.497938	0.469277	0.109521	0.097307	0.092154	0.168050	0.209050	0.237250	0.254774
81	0.497938	0.220808	0.110847	0.077198	0.100647	0.173900	0.189600	0.202300	0.250750
82	0.497938	0.389127	0.073696	0.065697	0.115603	0.172550	0.211300	0.206050	0.262050
83	0.497938	0.433789	0.094335	0.073144	0.102266	0.144103	0.209950	0.207050	0.262000
84	0.497938	0.489479	0.132642	0.079718	0.104000	0.152550	0.204650	0.209550	0.229500
85	0.497938	0.383445	0.104513	0.088899	0.099333	0.166950	0.229350	0.223200	0.238200
86	0.497938	0.311251	0.071514	0.078864	0.127854	0.144700	0.187850	0.192500	0.272450
87	0.497938	0.497938	0.200460	0.078176	0.097201	0.167200	0.197950	0.236850	0.214750
88	0.497938	0.126802	0.091615	0.071598	0.102519	0.157150	0.218900	0.197800	0.210400
89	0.497938	0.284133	0.078313	0.074378	0.122396	0.155951	0.204400	0.258450	0.242300
90	0.497938	0.263541	0.084445	0.074474	0.119094	0.164650	0.212000	0.252000	0.238300
91	0.497938	0.206552	0.346690	0.081327	0.133461	0.163150	0.193400	0.230200	0.220550
92	0.497938	0.339319	0.293704	0.082960	0.109466	0.170152	0.200350	0.225300	0.256700
93	0.497938	0.489700	0.094769	0.073231	0.134813	0.168501	0.200800	0.233500	0.227301
94	0.497938	0.480842	0.060556	0.089541	0.119042	0.149800	0.217618	0.216000	0.247100
95	0.497938	0.497938	0.393344	0.073855	0.103714	0.165650	0.230300	0.221800	0.249750
96	0.497938	0.204836	0.092259	0.117796	0.121437	0.176050	0.208400	0.221200	0.237050
97	0.497938	0.329237	0.088501	0.091398	0.122708	0.171152	0.197050	0.221500	0.230000
98	0.497938	0.202468	0.178332	0.080041	0.097718	0.157453	0.213850	0.213200	0.251300
99	0.497938	0.391502	0.100656	0.066627	0.121337	0.190550	0.193400	0.219000	0.256550
100	0.497938	0.506742	0.390384	0.089585	0.107267	0.147502	0.197700	0.223700	0.247151

(i) Watts–Strogatz derivative model

	ρ								
	0	0.01	0.05	0.1	0.2	0.4	0.7	0.85	1
1	0.497938	0.429186	0.590336	0.349187	0.415288	0.453273	0.418817	0.378891	0.347945
2	0.497938	0.294689	0.405565	0.423925	0.432178	0.397659	0.388262	0.378460	0.333028
3	0.497938	0.512410	0.344447	0.547906	0.328375	0.401896	0.442068	0.328068	0.329187
4	0.497938	0.279716	0.503862	0.298342	0.362449	0.392907	0.389885	0.429846	0.317481
5	0.497938	0.358542	0.476968	0.523779	0.434795	0.423954	0.434840	0.432290	0.384513
6	0.497938	0.273181	0.431754	0.266788	0.374735	0.367390	0.470526	0.371414	0.365277
7	0.497938	0.214364	0.554673	0.372505	0.424693	0.407156	0.460327	0.440095	0.323086
8	0.497938	0.378823	0.326787	0.309554	0.374818	0.459541	0.417928	0.419648	0.384022
9	0.497938	0.403702	0.193133	0.318432	0.306682	0.427216	0.413691	0.422391	0.337955
10	0.497938	0.364346	0.251289	0.379062	0.408520	0.488756	0.425860	0.509317	0.454423
11	0.497938	0.378564	0.356745	0.391798	0.349195	0.443644	0.453828	0.385801	0.415525
12	0.497938	0.279648	0.262004	0.301960	0.402022	0.390295	0.467923	0.375725	0.416278
13	0.497938	0.657790	0.391432	0.296951	0.404511	0.419882	0.397783	0.468338	0.332809
14	0.497938	0.511337	0.538410	0.375405	0.322911	0.378465	0.458123	0.452509	0.372314
15	0.497938	0.375709	0.276582	0.311825	0.401798	0.405822	0.452377	0.428344	0.357645
16	0.497938	0.340112	0.335970	0.302162	0.368090	0.393736	0.428114	0.418138	0.371540
17	0.497938	0.237263	0.426219	0.474323	0.458991	0.400803	0.389563	0.473219	0.335828
18	0.497938	0.226255	0.442305	0.313740	0.343229	0.417591	0.427346	0.345365	0.357054
19	0.497938	0.371449	0.355022	0.309422	0.328002	0.445235	0.438258	0.419990	0.395822
20	0.497938	0.465342	0.638709	0.360136	0.338384	0.453478	0.440746	0.367331	0.386784
21	0.497938	0.375769	0.352680	0.492372	0.472115	0.392781	0.430571	0.392075	0.399595
22	0.497938	0.274228	0.421509	0.476545	0.437562	0.460698	0.448818	0.441889	0.388743
23	0.497938	0.366942	0.294927	0.397043	0.375220	0.416604	0.374597	0.352098	0.392066
24	0.497938	0.433093	0.549422	0.341123	0.386571	0.417270	0.445430	0.411116	0.347954
25	0.497938	0.238052	0.375469	0.257729	0.423718	0.448847	0.481826	0.457418	0.377768
26	0.497938	0.271043	0.400789	0.418009	0.399112	0.426629	0.411259	0.389663	0.366993
27	0.497938	0.454012	0.388998	0.249695	0.477690	0.407169	0.426183	0.473148	0.361159
28	0.497938	0.533554	0.293774	0.592460	0.356324	0.405469	0.430159	0.366442	0.427003
29	0.497938	0.348017	0.302754	0.664959	0.366318	0.414082	0.449535	0.418353	0.333405
30	0.497938	0.512362	0.491540	0.410146	0.365253	0.446044	0.426746	0.475305	0.336231
31	0.497938	0.528755	0.330242	0.295729	0.449067	0.419594	0.451365	0.368583	0.356444
32	0.497938	0.356427	0.286668	0.344914	0.352447	0.419821	0.449725	0.470029	0.407609
33	0.497938	0.391137	0.392711	0.354160	0.471673	0.452850	0.414510	0.322697	0.367667
34	0.497938	0.332682	0.290187	0.264398	0.346105	0.390660	0.434343	0.406095	0.336556
35	0.497938	0.424223	0.342780	0.322290	0.427277	0.428307	0.408309	0.429408	0.372272
36	0.497938	0.213354	0.341844	0.454018	0.429948	0.461886	0.394576	0.352395	0.362370
37	0.497938	0.541534	0.304802	0.311401	0.380223	0.468716	0.464775	0.414808	0.385362
38	0.497938	0.351207	0.450111	0.338445	0.588370	0.452751	0.412872	0.348829	0.458542
39	0.497938	0.412573	0.308561	0.296611	0.415134	0.406477	0.456167	0.477402	0.317952
40	0.497938	0.614394	0.361944	0.417241	0.335567	0.446023	0.473982	0.459490	0.384410
41	0.497938	0.359550	0.361690	0.327395	0.358570	0.411857	0.411738	0.444761	0.424230
42	0.497938	0.467706	0.419321	0.313888	0.435998	0.399094	0.436072	0.390801	0.333934
43	0.497938	0.520010	0.406785	0.388923	0.468134	0.426152	0.419723	0.442751	0.401399
44	0.497938	0.404162	0.350543	0.394570	0.371008	0.460297	0.445729	0.362271	0.259910
45	0.497938	0.237789	0.283582	0.345353	0.339452	0.422893	0.488530	0.451926	0.344685
46	0.497938	0.460740	0.399632	0.445722	0.383432	0.428578	0.434555	0.486814	0.401966
47	0.497938	0.417183	0.582809	0.414316	0.429017	0.435486	0.471726	0.400905	0.333146
48	0.497938	0.511350	0.272302	0.355836	0.384778	0.385895	0.412210	0.449070	0.398209
49	0.497938	0.422835	0.522871	0.396801	0.309117	0.390045	0.435430	0.414634	0.349079
50	0.497938	0.279835	0.371412	0.313316	0.471449	0.388222	0.447471	0.434263	0.382371

(i) Watts–Strogatz derivative model (continued)

	ρ								
	0	0.01	0.05	0.1	0.2	0.4	0.7	0.85	1
51	0.497938	0.587907	0.301453	0.432886	0.355751	0.459426	0.445700	0.435254	0.400859
52	0.497938	0.270818	0.316640	0.308219	0.455863	0.471106	0.427689	0.448200	0.356474
53	0.497938	0.357519	0.387004	0.288505	0.300906	0.419424	0.457875	0.406931	0.388377
54	0.497938	0.331864	0.243377	0.363476	0.329900	0.439880	0.353682	0.373393	0.355434
55	0.497938	0.263772	0.238122	0.359971	0.448217	0.353971	0.459578	0.405424	0.410177
56	0.497938	0.261021	0.423006	0.357486	0.327003	0.433255	0.435474	0.406153	0.333863
57	0.497938	0.423087	0.399934	0.472678	0.384556	0.544252	0.423339	0.448038	0.488706
58	0.497938	0.438376	0.261471	0.357527	0.341938	0.386593	0.400321	0.434322	0.369921
59	0.497938	0.385984	0.390290	0.325593	0.541705	0.468785	0.365050	0.362408	0.395437
60	0.497938	0.490264	0.496077	0.281750	0.462278	0.410168	0.409409	0.384380	0.388008
61	0.497938	0.282292	0.278077	0.552766	0.338979	0.348122	0.430426	0.391679	0.351323
62	0.497938	0.515142	0.365825	0.277280	0.388739	0.405693	0.541080	0.459880	0.430690
63	0.497938	0.304385	0.462881	0.340987	0.440971	0.490565	0.457892	0.369180	0.286170
64	0.497938	0.349414	0.322624	0.280237	0.465814	0.428848	0.386282	0.433676	0.385441
65	0.497938	0.334998	0.279905	0.264616	0.320051	0.434099	0.455636	0.415861	0.429663
66	0.497938	0.441549	0.274049	0.340604	0.486835	0.397884	0.436436	0.362759	0.374270
67	0.497938	0.438016	0.293718	0.280968	0.445337	0.433448	0.420142	0.373226	0.349355
68	0.497938	0.297323	0.299156	0.294108	0.383351	0.386446	0.425341	0.443400	0.335794
69	0.497938	0.440041	0.387436	0.474341	0.438465	0.424798	0.457381	0.469086	0.363501
70	0.497938	0.202514	0.418404	0.343990	0.362560	0.434219	0.411848	0.359994	0.306463
71	0.497938	0.259854	0.242260	0.435127	0.364406	0.411723	0.443833	0.423428	0.450079
72	0.497938	0.389428	0.299124	0.337528	0.408603	0.410393	0.417230	0.368920	0.358211
73	0.497938	0.329524	0.516126	0.613043	0.435964	0.426087	0.427557	0.357123	0.319991
74	0.497938	0.484857	0.562637	0.351826	0.381547	0.448567	0.431387	0.470320	0.391358
75	0.497938	0.421509	0.363417	0.457294	0.465500	0.430019	0.424053	0.339633	0.385340
76	0.497938	0.333518	0.364636	0.492297	0.387474	0.408166	0.421678	0.377458	0.368948
77	0.497938	0.374241	0.319213	0.267711	0.342237	0.469804	0.453198	0.447163	0.368619
78	0.497938	0.354486	0.500713	0.308382	0.388309	0.436180	0.393568	0.391443	0.433266
79	0.497938	0.501010	0.347763	0.389247	0.502595	0.414443	0.465772	0.404905	0.368678
80	0.497938	0.294168	0.347816	0.515593	0.470724	0.417125	0.480617	0.405984	0.363348
81	0.497938	0.372967	0.380380	0.361561	0.418973	0.414887	0.389607	0.433961	0.339563
82	0.497938	0.329278	0.358420	0.316182	0.421563	0.419593	0.438148	0.432977	0.326912
83	0.497938	0.458578	0.297608	0.371825	0.399710	0.406380	0.386087	0.389715	0.407557
84	0.497938	0.337554	0.369644	0.337829	0.560019	0.443219	0.379859	0.400430	0.306470
85	0.497938	0.532164	0.399925	0.424593	0.314109	0.508263	0.456101	0.368865	0.392312
86	0.497938	0.284055	0.245990	0.461765	0.382671	0.378063	0.481415	0.380565	0.365905
87	0.497938	0.418030	0.264554	0.295155	0.381928	0.422313	0.413869	0.369301	0.338167
88	0.497938	0.220759	0.430273	0.456614	0.323049	0.420010	0.405987	0.412098	0.426771
89	0.497938	0.488565	0.358380	0.320986	0.281607	0.412229	0.412052	0.385783	0.394035
90	0.497938	0.275474	0.326018	0.371187	0.381970	0.531611	0.496267	0.421752	0.401802
91	0.497938	0.401531	0.383792	0.471814	0.368199	0.431949	0.406225	0.397739	0.405336
92	0.497938	0.279808	0.334038	0.247628	0.409183	0.456664	0.457492	0.394896	0.347983
93	0.497938	0.202125	0.398716	0.429294	0.385975	0.410934	0.374570	0.422321	0.353468
94	0.497938	0.404760	0.573730	0.340469	0.384197	0.450831	0.452497	0.385662	0.368709
95	0.497938	0.326344	0.414951	0.297160	0.296596	0.389200	0.460336	0.431718	0.421258
96	0.497938	0.265452	0.300887	0.306158	0.410536	0.445643	0.406729	0.485732	0.383251
97	0.497938	0.448523	0.471863	0.271122	0.443979	0.446670	0.488775	0.374986	0.350308
98	0.497938	0.507954	0.253009	0.304357	0.401916	0.382695	0.463747	0.432792	0.348431
99	0.497938	0.264394	0.325026	0.421614	0.357515	0.438761	0.425403	0.418827	0.312964
100	0.497938	0.263297	0.305222	0.298022	0.345433	0.490912	0.388478	0.402987	0.348954
