# Supplementary material for: Market, power, gift, and concession economies: Comparison using four-mode primitive network models
Source: PLoS One. 2025 Aug 12;20(8):e0330174. doi: 10.1371/journal.pone.0330174 (PMC12342310; doi:10.1371/journal.pone.0330174)
Supplement: Attachment — Submitted filename: Response_to_Reviewers_0715.docx [file pone.0330174.s004.docx]

July 15, 2025

Academic Editor

*PLOS ONE*

Dear Editor and Reviewers:

Thank you for your suggestions and comments on our manuscript titled “Market, power, gift, and concession economies: Comparison using four-mode primitive network models.” We also appreciate the time and effort you and each of the reviewers have dedicated to providing insightful feedback on ways to strengthen our paper. We have incorporated changes that reflect the detailed suggestions you have so graciously provided. We hope that our edits and the responses provided below satisfactorily address all the issues and concerns you and the reviewers have noted.

To facilitate your review of our revisions, the following is a point-by-point response to the questions and comments delivered in your letter [PONE-D-25-19624].

**Response to Editor:**

1. We invite you to submit a revised version of the manuscript that addresses the points raised during the review process.

**Response:** Thank you for your invitation. We revised the manuscript, considering the reviewers’ suggestions.

2. Include the following items when submitting your revised manuscript: ‘Response to Reviewers,’ ‘Revised Manuscript with Track Changes,’ and ‘Manuscript’ without tracked changes.

**Response:** Following your instruction, we have submitted three items.

3. Submit your revised manuscript by Jun 26 2025 11:59PM. If you will need more time than this to complete your revisions, please reply to this message or contact the journal office.

**Response:** I contacted the journal office and Editorial Assistant, Rumelda Clarisse Magallanes, informed me that the deadline has been extended to 26 September 2025.

4. If you would like to make changes to your financial disclosure, please include your updated statement in your cover letter.

**Response:** There are no changes to our financial disclosure.

5. Guidelines for resubmitting your figure files are available below the reviewer comments at the end of this letter.

**Response:** We have resubmitted our figure files using the digital diagnostic tool PACE.

**Response to Journal Requirements:**

1. Ensure that your manuscript meets *PLOS ONE*’s style requirements.

**Response:** We have checked the style requirements and have ensured that these are met.

2. Please provide a complete Data Availability Statement in the submission form.

**Response:** We have written: “All data are present in the manuscript and supporting information files” as our Data Availability Statement.

**Response to Reviewer #1:**

1. This manuscript proposes a novel comparison of four economic modes—market, power, gift, and concession—using network models inspired by the theories of Polanyi, Karatani, and Graeber. The topic is original and interdisciplinary, and the attempt to formalize economic systems via network metrics is commendable. However, the paper in its current form requires substantial revision before it can be considered for publication.

**Response:** Thank you for your evaluation. We have revised and supplemented our manuscript as follows.

2. Conceptual Clarity: Key terms such as “concession economy” and “We-turn philosophy” lack clear definitions. Their theoretical grounding and practical implications should be elaborated. The relationships among the four economic modes (e.g., whether they are mutually exclusive, overlapping, or sequential) should also be clarified.

**Response:** To clarify the differences between the four modes, we have added a description of “concession economy” and the other three economic modes (pp. 4–5, lines 95–118). We have added some descriptions and references on “We-turn philosophy” and its economic perspective (pp. 7–8, lines 160–187, and references [18,19]). Further, we have added a description regarding the relationships among the four economic modes (pp. 5–6, lines 119–133). Note that the page and line numbers are from “Revised Manuscript with Track Changes.”

3. Modeling Details: The paper does not provide enough information about how the network models are constructed. What are the assumptions, node/edge definitions, simulation parameters, or data sources? These need to be explained clearly.

**Response:** We have added a description of the definition of nodes/edges (p. 11, lines 280–282), and detailed definitions, simulation parameters, codes, and data are provided in S1 Code and S2 Data as Supporting Information.

4. Interpretation of Results: The conclusions (e.g., “market economy leads to inequality,” “concession economy is ideal”) are too strong given the abstract nature of the models. The authors should avoid overgeneralization and provide more balanced, evidence-based discussion.

**Response:** We have rewritten the points raised and have tried to provide a more balanced discussion overall in our manuscript (p. 2, lines 31–33, p. 6, lines 144–145).

5. Language and Structure: The manuscript requires careful language editing to improve clarity and flow. Some concepts are densely presented or ambiguously phrased.

**Response:** As written in response to #2 above, to clarify the differences between the concepts of the four econimic modes and the We-turn philosophy, we have added descriptions that distinguish between them. Further, we have had our paper edited by and English editing company to improve the language, syntax, flow, and clarity.

**Response to Reviewer #2:**

1. The manuscript presents an interesting and highly conceptual framework grounded in philosophical theories such as “We-turn” and “baseline communism.” While the integration of these ideas into a network model is commendable, the manuscript lacks sufficient clarity and rigor in the definition of key modeling assumptions. Specifically, the mathematical encoding of core concepts such as “enclosure” and “de-obligation” within the network formation process is not clearly articulated. Additionally, the implementation of critical dynamics—such as edge deletion procedures (e.g., the “oblivion” mechanism in the concession economy)—remains vague. Greater technical transparency, including algorithmic steps or pseudocode, would significantly improve the model’s reproducibility.

**Response:** Thank you for your evaluation. To enhance technical transparency and reproducibility, we have provided the mathematical code and algorithms as Supporting Information in S1 Code.

2. The interdisciplinary ambition of the manuscript is appreciated, particularly its philosophical framing of economic modes. However, from a technical modeling perspective, the novelty is limited. The use of a simple random graph model (inspired by Kauffman’s button and thread model) does not substantially advance existing literature in network simulations. kindly refer:

(1) <https://doi.org/10.1002/dac.6105>, (2) <https://doi.org/10.1038/s41598-024-66515-x>,

(3) <https://doi.org/10.1080/14786451.2024.2364226>, (4) <https://doi.org/10.1186/s44147-024-00460-4>, (5) <https://doi.org/10.1016/j.icte.2024.05.002>, (6) [https://doi.org/10.1016/j.heliyon. 2024.e32217](https://doi.org/10.1016/j.heliyon.%202024.e32217), (7) <https://doi.org/10.1016/j.eij.2024.100577>, (8) <https://doi.org/10.1002/dac.6105>, (9) <https://doi.org/10.1002/eng2.13019>, (10) <https://doi.org/10.18280/mmep.110801>, (11) [https:// doi.org/10.1002/dac.5901](https://doi.org/10.1002/dac.5901).

**Response:** Thank you for your suggestion. As you pointed out, we used a simple random graph model, but we believe that it is novel to distinguish between the four economic modes and model each of them based on this model. Furthermore, as described in #3 below, we believe that it is also novel that the positioning of the four modes was clarified by comparing them with more structurally nuanced models. Thank you for introducing the existing literatures. After reading these, we confirmed that (1)(2)(5)(6)(7)(8)(9)(11) are related to antenna design, (10) is related to harmonic bandpass filter design, (3) is related to photovoltaic/thermal performance prediction, and (4) is related to security measures for medical systems. These literatures mention neural networks, communication networks, and medical networks, but we considered them difficult to use as references for building network models for economic modes.

(1) A combined approach of pattern recognition using convolutional neural networks and relational learning using graph neural networks for the design optimization of frequency-selective surface antennas.

(2) Design optimization of a wideband dual mode dual-polarized antenna for 5G wireless communication networks using characteristic mode analysis.

(3) Photovoltaic/thermal performance prediction using machine learning algorithms in artificial neural network.

(4) Improving security in medical network systems based on the Medical Internet of Things by using a stateless mechanism and symmetric key management scheme.

(5) Design of a broadband patch antenna for satellite communications using a metamaterial with a split ring resonator structure.

(6) Design of a dual-mode, dual-polarized antenna for GSM and 5G wireless communications using characteristic mode analysis.

(7) Improved brain tumor classification performance using convolutional neural network-based brain tumor classification and growth distribution depth using a survival analysis model.

(8) Same as (1).

(9) Design and demonstration of viability and advantages of a dual-band circular fractal antenna for Wi-Fi applications.

(10) Design of a tiny stepped impedance resonator that enables higher rejection band and miniaturization of size for bandpass filters to suppress harmonics.

(11) Design optimization of multi-input multi-output antennas for S-band satellite communication networks.

3. The absence of comparison with more established and structurally nuanced models—such as Exponential Random Graph Models or agent-based models—reduces the strength of the manuscript’s methodological contribution.

**Response:** Regarding representative random graph models that are more established and structurally nuanced models, we have taken up the Watts–Strogatz model, which has small-world properties, and the Barabási–Albert model, which has scale-free properties, and compared them with the four economic mode models (p. 9, lines 225–230, p. 10, lines 258–260, pp. 13–14, lines 342–360, pp. 23–28, lines 551–673, p. 29, lines 699–708). Additionally, we have added content detailing literature related to this study regarding agent-based models (p. 28, lines 674–685, and reference [38]).

4. The modeling approach relies on uniform edge generation probabilities across the different economic systems. This assumption may not adequately capture the structural and behavioral differences that the manuscript seeks to explore. While the use of metrics such as the Gini coefficient, assortativity, and centrality is appropriate, their application alone does not sufficiently support the manuscript’s broader philosophical and normative claims—particularly regarding the supposed feasibility or superiority of concession economies. Moreover, the model lacks sensitivity analysis or robustness checks to assess how outcomes vary with parameter changes.

**Response:** As written in response to #3 above, we have emplyed the Watts–Strogatz model and the Barabási–Albert model as models that do not have uniform edge generation probabilities. Additionally, by using the rewiring probability parameter of the Watts–Strogatz and its derivative models as a sensitivity analysis, we have presented a relationship between the Watts–Strogatz, its derivative models, gift economies, and concession economies (pp. 27–28, lines 646–673).

5. The results are presented primarily through descriptive plots, without statistical validation or inferential analysis. To strengthen the manuscript's conclusions, the authors should consider including confidence intervals, standard deviations, or hypothesis tests. At present, some interpretations in the discussion—such as asserting the practical viability of the concession economy—appear speculative and are not adequately substantiated by the simulation results alone. The absence of detailed statistical summaries across multiple runs further limits the reliability of the findings.

**Response:** To strengthen statistical verification, we have performed 100 simulations for each of the four economic modes, the Watts–Strogatz model (and derivative), and the Barabási–Albert model, and have presented the results in box-and-whisker plots, correlation graphs (correlation coefficients and coefficients of determination), and graphs with error bars of standard deviations (pp. 24–28, lines 571–673, Figs 6–9). These data are listed in S2 Data as Supporting Information.

6. While the authors describe the model as primitive, the study would benefit greatly from some form of empirical validation. Even a preliminary comparison with real-world data or stylized facts from socio-economic systems would improve the manuscript’s credibility. Without empirical grounding, it is difficult to assess whether the observed model dynamics have practical significance or are merely artifacts of the simulation assumptions.

**Response:** In addition to our existing text at the end of the discussion outlining the limitations of the simulation, we have added descriptions and references regarding empirical and historical evidence (pp. 30–31, lines 732–752, and references [41–43]).

Thank you once again for giving us the opportunity to strengthen our manuscript with your valuable comments and queries. We have worked hard to incorporate your feedback and hope that these revisions persuade you to accept our submission.

Sincerely,

Takeshi Kato

Hitachi Kyoto University Laboratory, Open Innovation Institute, Kyoto University

Yoshidahonmachi, Sakyo-ku, Kyoto-shi, Kyoto 606-8501, Japan

+81-75-753-9716

kato.takeshi.3u@kyoto-u.ac.jp
